# From Local Atomic Structure to X‑ray Spectra: Absorber-Centric Machine-Learning Encoding

**DOI:** 10.1021/acs.jpca.6c01127

**Published:** 2026-05-06

**Authors:** Thomas James Pope, Bowen Li, Hendrik Junkawitsch, Annika Bande, Thomas James Penfold

**Affiliations:** † Chemistry − School of Natural and Environmental Sciences, Newcastle University, Newcastle upon Tyne NE1 7RU, U.K.; ‡ Research Software Engineer Group, 5994 Newcastle University, Newcastle upon Tyne NE1 7RU, U.K.; § Helmholtz-Zentrum Berlin für Materialien und Energie GmbH, 28340 Theory of Electron Dynamics and Spectroscopy, Hahn-Meitner-Platz 1, Berlin 14109, Germany; ∥ Institute for Computer Science, Humboldt-Universitát zu Berlin, Unter den Linden 6, Berlin 10099, Germany; ⊥ Leibniz University Hannover Institut of Inorganic Chemistry, Callinstr. 9, Hannover 30167, Germany

## Abstract

X-ray spectroscopy provides sensitive, element-specific insight into local geometric and electronic structures, but predictive first-principles simulations can be computationally expensive for large and chemically diverse molecular systems. Recent machine-learning approaches have shown promise in accelerating structure-to-spectrum prediction; however, most directly regress discretized spectral intensities and rely on hand-crafted geometric descriptors centered on the absorbing atom. Herein, we introduce a machine learning framework that encodes a detailed, environment-aware representation of the nuclear structure beyond the absorbing site. The model combines these descriptors with a physically motivated, multiscale Gaussian spectral basis whose coefficients are obtained via ridge projection, enforcing smoothness and spectral consistency. To further enhance robustness across chemical and conformational diversity, we employ a multiscale structural similarity loss that couples geometric and spectral resolution. This integrated approach yields accurate and transferable predictions across a wide range of molecular geometries and chemical environments while maintaining physical interpretability. The proposed framework establishes a physically structured and scalable route to machine-learned X-ray spectroscopy.

## Graphical abstract

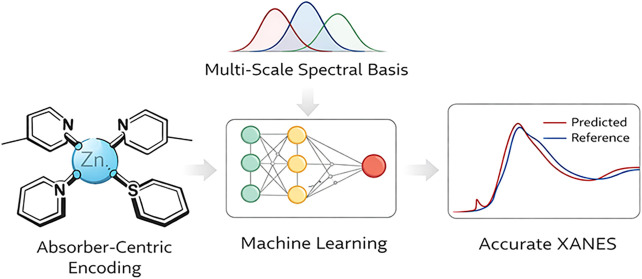

## Introduction

X-ray spectroscopy is a powerful and widely applied technique for probing local coordination environments, oxidation states, and electronic structure in chemical, catalytic, and materials systems.^1–5^ Its impact continues to expand with the development of next-generation synchrotron and free-electron laser facilities,^6–11^ alongside increasingly accessible laboratory-based instrumentation,^12–14^ enabling more precise and routine measurements across both fundamental research and applied technologies.

Alongside these experimental advancements, computational X-ray spectroscopy has progressed significantly, driven by the need for fast and accurate interpretation of measured spectra.^15–17^ A broad range of first-principles methods is now available, spanning both *ab initio* approaches^18–23^ and density functional theory (DFT)-based techniques.^24–27^ These developments have not only improved the fidelity of spectral predictions but have also deepened our understanding of the underlying physical mechanisms that shape experimental observables, such as the factors determining X-ray spectral lineshapes. Moreover, the increasing availability of large and systematically generated computational data sets has opened new opportunities for data-driven and machine-learning frameworks to augment traditional computational spectroscopy approaches.^28–30^

In the machine-learning of spectroscopic observables, forward-mapping from structure to spectrum most closely aligns with the central objective of computational chemistry and provides a natural bridge between experiment and theory. As a result, there has been substantial interest in developing efficient and accurate models for X-ray spectroscopy,^31–51^ including efforts focused on interpretability.^53–55^ Among these approaches, the XANESNET framework^56,57^ has been applied to both K-edge XANES and valence-to-core X-ray emission spectroscopy,^58^ demonstrating that meaningful chemical insight^59–61^ can be extracted from the predictions. Despite this progress, two challenges hinder improvements in performance. First, many existing models directly regress spectral intensities on a discretized energy grid, requiring high model capacity to reproduce inherently smooth spectral features and often resulting in limited robustness and transferability. Second, local structural environments are typically described using manually parametrized symmetry functions centered exclusively on the absorbing atom, excluding broader environmental information and restricting the expressive power of the representation.

In this work, we seek to address both limitations by introducing a physically structured representation of both molecular geometry and spectral line shape into XANESNET.^56^ Information summarizing the local neighborhood of the absorbing atom is introduced using a radial encoder in which the centers and widths of radial shells are learnable parameters. Rather than imposing fixed coordination shells, the model infers smooth radial weighting functions that adapt to the chemically relevant bonding distances. To represent spectra, we project each profile onto a multiscale Gaussian basis using ridge-regularized coefficient extraction. This yields a compact and smooth spectral representation that separates sharp near-edge features from the broader background structure while remaining readily transferable to experimental data. Using this approach combined with a multiscale structural similarity loss, we demonstrate a substantial improvement compared to the performance of previous models.

## Method and Computational Details

### Data Sets

The reference data sets used in this work were introduced in refs 57,58 and are publicly available at ref 62. They consist of molecular geometries (“samples”) of first-row transition metal complexes (Ti–Zn) extracted from the transition-metal Quantum Machine (tmQM) data set.^63,64^ Full details of the construction, spectral widths, and composition of the tmQM data set are provided in refs 57,58 .

For X-ray absorption spectroscopy (XAS), K-edge XANES spectra were computed using multiple scattering theory (MST) as implemented in the FDMNES package,^65^ following the procedure described in ref 57. For X-ray emission spectroscopy (XES), K-edge valence-to-core (VtC) XES spectra were calculated using a quasi-one-electron approach^27^ implemented in ORCA,^66^ employing the TPSSh exchange–correlation functional^67,68^ and the def2-SVP basis set.^69^

In total, 18 independent data sets were constructed, corresponding to each first-row transition metal (Ti–Zn) for both the XAS and XES edges. The size of the data sets ranges from approximately 1100 samples (V) to 8,660 (Ni). For each, 20% of samples were randomly selected to form a held-out test set used exclusively for model evaluation.

### Deep Neural Network

#### Spectral Representation

To obtain a compact and smooth representation, spectra are expanded in a Gaussian spectral basis **Φ** ∈**R** ^N_*E*_×K^ defined over the discrete energy grid *E*, where *N*_*E*_ denotes the number of energy grid points and *K* the total number of spectral basis functions.

Each column **ϕ**_*k*_(*E*) corresponds to a Gaussian basis function defined as

1 $$\phi_{k}\left(E\right)=\exp\left[-\frac{1}{2}{\left(\frac{E-\mu_{k}}{w_{k}}\right)}^{2}\right]$$

centered at energy μ_*k*_ with width *w*_*k*_. The basis matrix is constructed by evaluating each basis function on the discrete energy grid

2 $$\Phi_{\mathrm{ik}}=\phi_{k}\left(E_{i}\right),i=1,\cdot \cdot \cdot ,N_{E},\;k=1,\cdot \cdot \cdot ,K$$

To capture spectral features across multiple length scales, several Gaussian width groups are employed. Throughout the present work, four Gaussian functions are placed at each energy grid point with widths

3 $$\sigma_{k}\in \left\{0.5,1.0,2.0,4.0\right\}\mathrm{eV}$$

This multiscale basis enables the simultaneous representation of sharp near-edge structure and broader spectral features.

Given the basis matrix **Φ** and regularization parameter λ > 0, the ridge-regularized coefficients are obtained using

4 $$A={\left(\Phi^{Τ}\Phi +\lambda I_{K}\right)}^{-1}\Phi^{Τ}$$

where **I**_*K*_ is the *K* × *K* identity matrix. This operator corresponds to the ridge-regularized pseudoinverse of the basis matrix and stabilizes the coefficient estimation in the presence of basis redundancy. The application of this operator transforms any spectrum directly into its coefficient representation

5 $$c^{*}=\mathrm{Ay}$$

where c* ∈ **R** ^*K*^ contains the ridge coefficients associated with the Gaussian basis. These coefficients provide a compact and smooth decomposition of the spectral profile, quantifying the contribution of each basis component to the observed absorption spectrum. The coefficients **c** are obtained from the reference spectra via a least-squares projection onto the Gaussian basis. While this projection is not strictly lossless, the chosen basis is sufficiently dense and smooth that the reconstruction error is negligible for the spectra considered here.

#### Geometric Representation and Environment Encoder

In this section, we describe our approach for encoding the molecular environment into a geometric representation, which is illustrated schematically in Figure 1. For a molecule containing *N* atoms, we compute a two-dimensional descriptor represented as a tensor of shape (*N*, *d*), where each row corresponds to a feature vector associated with a single atom. In the present work, this per-atom descriptor is obtained using one of two approaches. The first is a two-layer MACE representation, defined by the atomic feature vectors produced after each message-passing block of a MACE-MP0 model.^70,71^ The second approach employs weighted atom-centered symmetry functions (wACSFs), computed from the perspective of every atom in the molecule. In both cases, the resulting per-atom feature vectors are denoted as

6 $$x=\left[x_{0},x_{1},\cdot \cdot \cdot ,x_{N-1}\right],x_{i}\in R^{d}$$

where **x**_0_ corresponds to the atomic feature vector of the absorbing atom, only one is defined for each input. In addition, we compute the Euclidean distance from each atom to the absorbing atom, resulting in a distance vector of length *N*.

**Figure 1:**
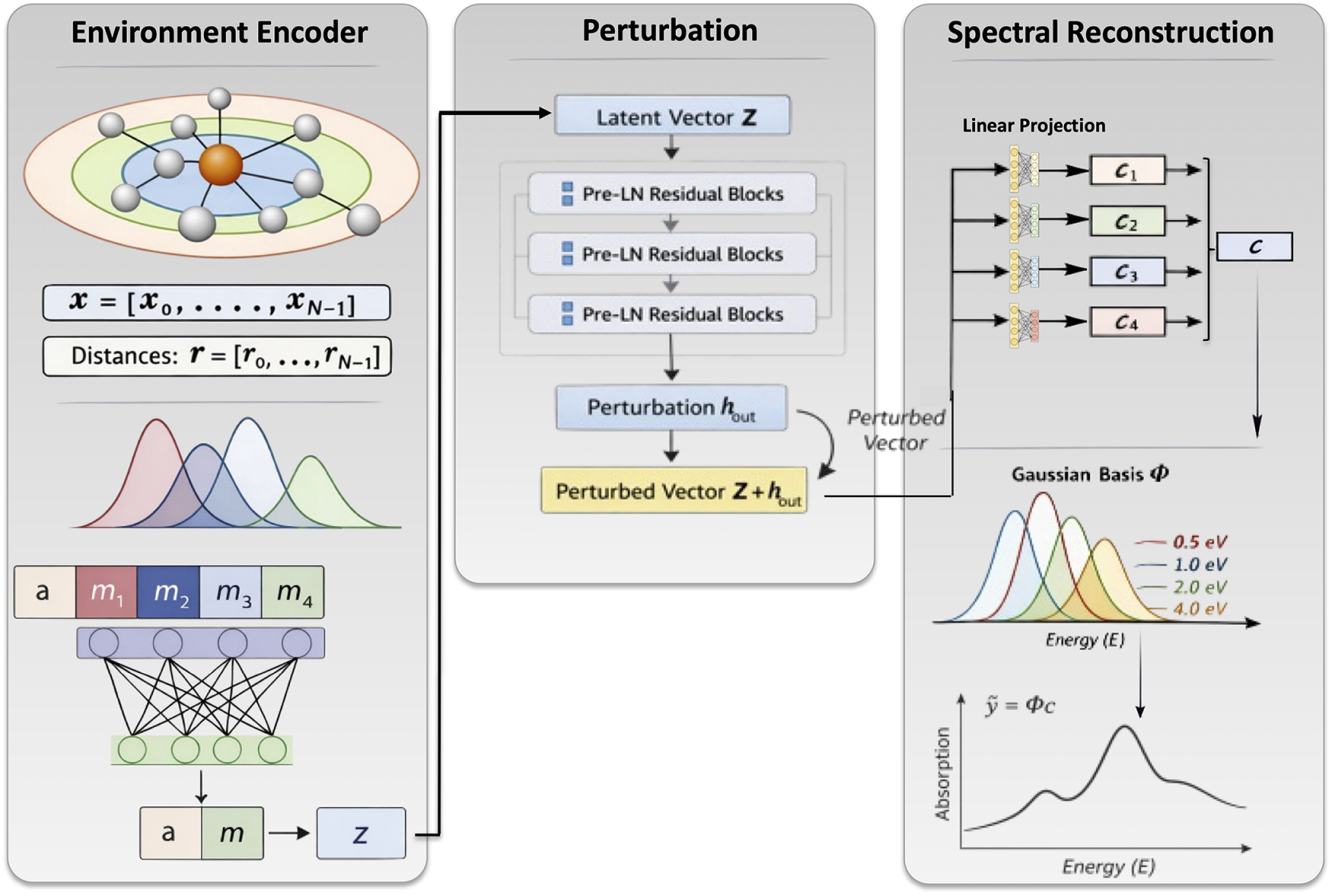
Schematic overview of the workflow proposed in this work. A molecule containing *N* atoms is first encoded as a two-dimensional per-atom descriptor. The descriptor associated with the absorbing atom is retained explicitly, while the descriptors of the remaining *N* – 1 atoms are aggregated into an environment representation, as described in the main text. These components are then concatenated and processed by prelayer-normalized residual multilayer perceptron (MLP) blocks to form a final molecular descriptor. This descriptor is subsequently passed through a linear projection layer to predict the coefficients of the Gaussian spectral basis corresponding to the X-ray spectrum. Model performance is assessed using a composite loss function comprising a multiscale structural similarity index measure and a mean-squared-error term on the Gaussian coefficients.

The encoder defines a list of learnable soft radial shells centered on the absorbing atom with radius *r*_s_ and blur-factor (width), *b_s_*, not to be confused with basis width *w_k_*. A shell descriptor is computed with a weighted sum of the atomic descriptors

7 $$m_{s}=\sum_{i=1}^{N-1}w_{\mathrm{is}}x_{i}$$

Each atom’s contribution to a shell is weighted by a Gaussian centered on the shell radius

8 $${\mathrm{w̃}}_{\mathrm{is}}=\exp\left[-\frac{1}{2}{\left(\frac{r_{i}-r_{s}}{b_{s}}\right)}^{2}\right]$$

normalized across atoms as

9 $$w_{\mathrm{is}}=\frac{{\mathrm{w̃}}_{\mathrm{is}}}{\sum_{j}{\mathrm{w̃}}_{\mathrm{js}}+\varepsilon }$$

For each shell *s*, we compute a weighted mean of neighbor features

10 $$m_{s}=\sum_{i=1}^{N-1}w_{\mathrm{is}}x_{i}$$

The shell summary is formed by concatenating each shell summary, so the total length is *d* × *S*, where *d* is the length of the input descriptor for each atom. This is passed through a linear projection that fuses the per-shell features into a single feature vector of size *d*. This shell summary is then fused with absorber embedding, i.e., the atomic feature vector of the absorbing atom (**x**_0_), forming a latent vector, **z**.

The latent representation is refined through a stack of *L* prelayer-normalized residual MLP blocks. Starting from the initial latent vector **h** ^(0)^ = **z**, each block applies a transformation of the form

11 $$h^{\left(l+1\right)}=h^{\left(l\right)}+W_{2}^{\left(l\right)}\phi \left(W_{1}^{\left(l\right)}\mathrm{LN}\left(h^{\left(l\right)}\right)\right)$$

where **W**_1_^(l)^ and **W**_2_^(l)^ are learnable weight matrices, ϕ denotes a nonlinear activation function (GeLU), and LN is layer normalization. A final layer normalization is applied

12 $$h_{\mathrm{out}}=\mathrm{LN}\left(h^{\left(L\right)}\right)$$

This residual formulation preserves the original latent signal while allowing nonlinear refinement, improving optimization stability, and preventing feature collapse.

Subsequently, **h**_out_ is used as an input for *K* groups of coefficients, each corresponding to one Gaussian width bin, and passed through a final linear projection layer. The output of each head therefore provides the predicted coefficients associated with its respective Gaussian width over all energy grid points. Combining the encoder and the multihead projection with the spectral basis yields the overall end-to-end mapping

13 $$ŷ=\Phi f_{\mathrm{head}}\left(f_{\mathrm{enc}}\left(x,r\right)\right)$$

where Φ denotes the fixed spectral basis. The full model implementation, along with all other methods used in this work, is available publicly within the XANESNET software package.^56^

#### Models, Training Details, and Hyperparameters

Throughout this work, we use the XANESNET software.^56^ This is programmed in Python 3 with Pytorch.^72^ The atomic simulation environment API^73^ is used to handle and manipulate molecular structures. The code is publicly available under the GNU Public License (GPLv3) on GitLab.^56^ For the model described above, there are a number of model hyperparameters that need to be defined. These have been optimized to provide the most consistent performance across the training sets used herein.

All spectra are represented using a Gaussian basis expansion with four widths: 0.5, 1.0, 2.0, and 4.0 eV. Basis functions are placed at every fourth point on the energy grid. For the learnable soft radial embedding, we use four radial shells with a maximum cutoff radius of 7.0 Å. The prelayer-normalized residual MLP contains a single block (*L* = 1), consisting of two hidden layers with Gaussian Error Linear Unit (GeLU) activations and dropout applied with probability *p* = 0.1. The size of the resulting model (i.e., number of free parameters) depends on the length of the input descriptor, and therefore, when the MACE descriptor is used, the number of trainable parameters is 12 × 10^5^, while when the wACSFs descriptor is used, this reduces to 3 × 10^5^. Below, these models are denoted as the AWE-wACSFs or AWE-MACE models, where AWE denotes absorber with environment.

In addition to the models described above, performance is benchmarked against a multioutput multilayer perceptron (MLP) model previously reported in refs 57,58 . These reference models consist of an input layer with *N* neurons, which accepts a feature vector of length *N* encoding the local environment of the absorbing atom only, followed by two hidden layers of 512 neurons each. The output layer directly regresses the discretized X-ray spectrum. For these models, the total number of trainable parameters is approximately 7 × 10^5^ when using wACSF descriptors and 8 × 10^5^ when using MACE descriptors. In the following text, these are referred to as AO-wACSFs and AO-MACE models, where the AO denotes absorber only.

Throughout training, all model parameters **W** are optimized via iterative forward and backward propagation to minimize the empirical loss function *J*(**W**). In all models, we directly optimize a multiscale structural similarity index measure (MS-SSIM) that compares predicted and reference spectra. For the environment-encoder models, an additional mean-squared-error (MSE) loss is applied to the Gaussian basis coefficients. The total loss function is therefore defined as

14 $$L^{*}=L_{\mathrm{SSIM}}+\mathrm{MSE}\left(c_{\mathrm{predicted}},c_{\mathrm{reference}}\right)$$

where the first term enforces spectral fidelity, while the second penalizes deviations between the predicted and reference Gaussian basis–set coefficients. The spectral similarity term is formulated using a one-dimensional MS-SSIM, evaluated over a set of *K* = 3 Gaussian-weighted windows with widths {*w*_*k*_} of 3%, 5%, and 7% of the total spectral length, chosen to correspond with the average widths of the peaks in the spectra. The two terms act on complementary representations of the spectrum. In practice, these terms are naturally balanced due to their normalization, and we find that introducing additional weighting factors does not lead to systematic improvements.

For a given scale *k*, the single-scale structural similarity index between the predicted spectrum ŷ and the reference spectrum *y* is defined as

15 $${\mathrm{SSIM}}^{\left(k\right)}\left(ŷ,y\right)=\frac{\left(2\mu_{ŷ}^{\left(k\right)}\mu_{y}^{\left(k\right)}+C_{1}\right)\left(2\sigma_{ŷy}^{\left(k\right)}+C_{2}\right)}{({\left(\mu_{ŷ}^{\left(k\right)}\right)}^{2}+{\left(\mu_{y}^{\left(k\right)}\right)}^{2}+C_{1})\left({\left(\sigma_{ŷ}^{\left(k\right)}\right)}^{2}+{\left(\sigma_{y}^{\left(k\right)}\right)}^{2}+C_{2}\right)}$$

where μ_*ŷ*_^(*k*)^ and μ_*y*_^(*k*)^ denote the local means of the predicted and reference spectra, respectively, obtained by convolution with a Gaussian kernel of width *w*_*k*_. The quantities σ_*ŷ*_^(*k*)^ and σ_*y*_^(*k*)^ are the respective local standard deviations, and σ_*ŷy*_^(*k*)^ is the local cross-covariance. The global data range is defined as

16 R=max(ŷ,y)−min(ŷ,y,0)

and is used to define the stabilization constants

17 $$C_{1}={\left(0.01R\right)}^{2},,C_{2}={\left(0.03R\right)}^{2}$$

which ensures numerical stability in regions of low signal variance. The prefactors of 0.01 and 0.03 were found to give good stability. The scalar similarity score *s*_*k*_ at each scale is obtained by averaging SSIM^(k)^ over all spectral points.

The multiscale structural similarity is then computed as a weighted average across all scales

18 $$\mathrm{MS}‐\mathrm{SSIM}\left(ŷ,y\right)=\sum_{k=1}^{K}{\mathrm{\omega ̃}}_{k}s_{k},{\mathrm{\omega ̃}}_{k}=\frac{\omega_{k}}{\sum_{j=1}^{K}\omega_{j}}$$

where {ω_*k*_} are user-defined scale weights, taken to be uniformly normalized in this work. Finally, the spectral reconstruction loss is defined as

19 $$L_{\mathrm{MS}‐\mathrm{SSIM}}=1-\mathrm{MS}‐\mathrm{SSIM}\left(ŷ,y\right)$$

so that minimization of *L*_MS‑SSIM_ promotes agreement between predicted and reference spectra in terms of both the global spectral envelope and fine-scale structural features across multiple length scales.

Gradients of the loss with respect to the internal weights were estimated over minibatches of 32 samples and iteratively updated according to the adaptive moment estimation (ADAM) algorithm. An annealed learning rate was used throughout, with the learning rate initially set to 2 × 10^–3^, then reduced by a factor of 2 every 100 epochs. Internal weights were initially set according to ref 74. Unless explicitly stated in this Article, optimization was carried out over 1000 iterative cycles through the network (commonly termed epochs).

## Results and Discussion

We now turn to the Results and Discussion, which are organized into three main parts. First, we evaluate the model performance across all nine first-row transition-metal databases for both XAS and XES spectra, demonstrating its broad applicability across systems. These results are benchmarked against previously published machine-learning studies to establish a robust performance baseline. Next, we extend the analysis to quantify predictive uncertainty and to elucidate the interpretability of the model, providing insight into how structural and electronic features are encoded in the learned representations. Finally, we apply the framework to a series of representative molecular case studies, illustrating its capability to deliver physically meaningful predictions and to uncover structure–spectra trends.

### Performance

We first evaluate the performance of the different descriptor–model combinations for both X-ray absorption (XAS) and X-ray emission (XES) across the nine transition-metal systems considered in this work. Models based solely on descriptors of the absorbing atom are denoted as absorber-only (AO), whereas models that additionally incorporate the radially encoded chemical environment are denoted as absorber with environment (AWE). Figure 2 summarizes the results using box plots, showing the distribution of prediction errors for each model–element combination. The final column (“ALL”) corresponds to a unified model trained on the aggregated data set comprising all nine transition-metal systems.

**Figure 2:**
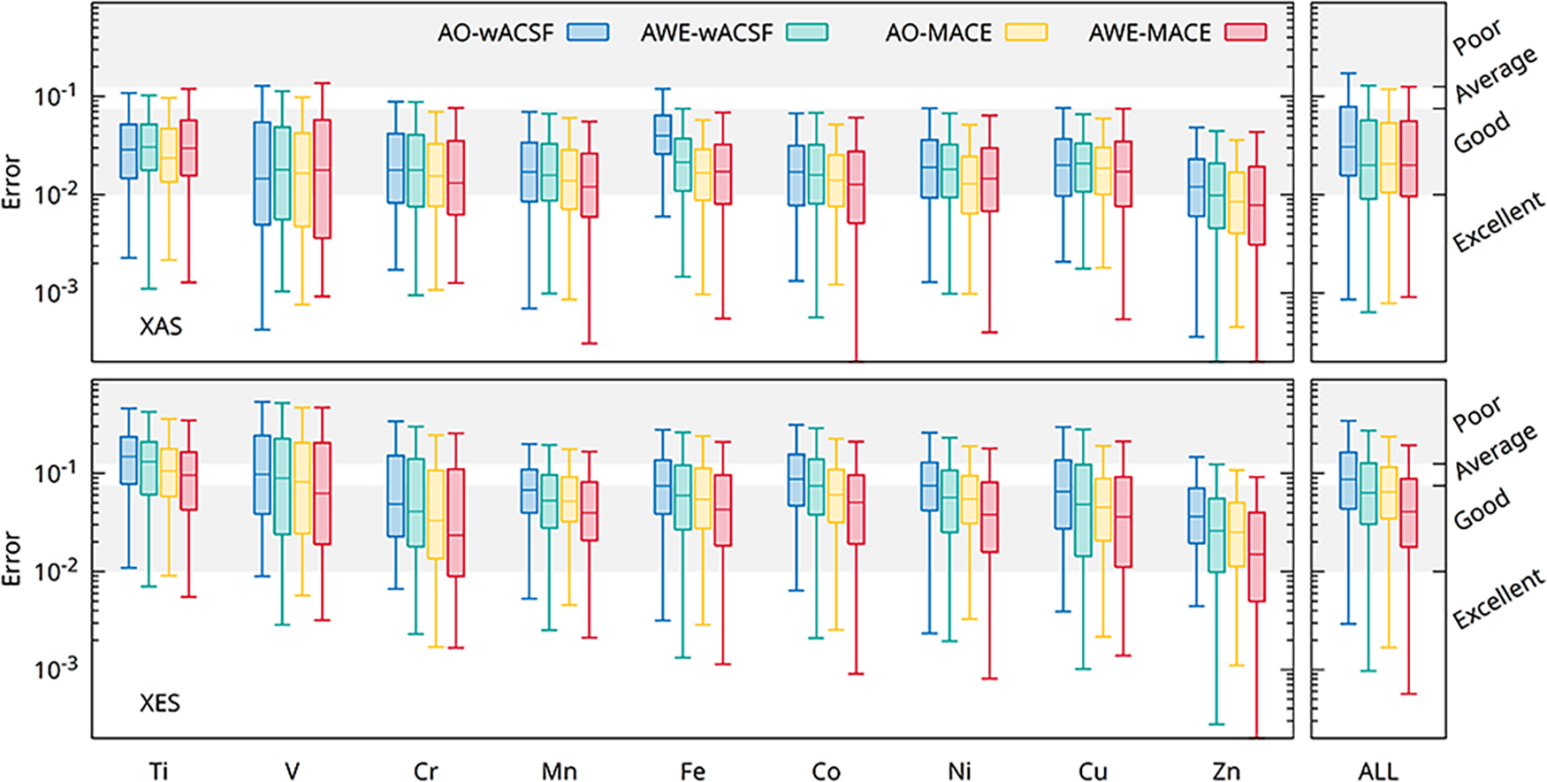
Box plots illustrating the predictive performance of the AO-wACSF (blue), AWE-wACSF (green), AO-MACE (yellow), and AWE-MACE (red) models for XAS (upper) and XES (lower) across the nine transition-metal (TM) K-edges considered in this work, as well as for the model trained simultaneously on the combined data set (“ALL”). A comprehensive numerical summary of the performance metrics for all models and elements is provided in Tables 1 and S1.

The prediction errors reported throughout are quantified using the same multiscale structural similarity (MS-SSIM) metric employed as the training loss function, ensuring direct consistency between model optimization and evaluation. As a practical reference, MS-SSIM values below 0.01 indicate excellent agreement between predicted and reference spectra, values below 0.075 correspond to good agreement, values in the range of 0.075–0.125 denote moderate agreement with noticeable deviations in peak positions and intensities, and values exceeding 0.125 generally reflect poor spectral fidelity. For clarity, these threshold regions are indicated on all plots. A comparison of the MS-SSIM loss against MSE is shown in Figures S1 and S2.

Across both spectral modalities (XAS and XES), broadly consistent trends are observed across the transition-metal series. In general, prediction errors decrease toward the later elements. This reflects a combination of data set and physical effects: later transition metals are typically represented by larger and more diverse training sets (see Table S1), while the progressive filling of the d-manifold leads to reduced diversity in oxidation states and coordination environments, as well as weaker metal–ligand hybridization. Together, these factors yield spectral signatures that are structurally and electronically less complex and easier to learn.

Although less pronounced for the early elements in XAS (notably Ti and V), a systematic improvement is observed when moving from AO-wACSF to AO-MACE, followed by further gains upon the explicit inclusion of the local chemical environment in the AWE models. As summarized in Table 1, for XAS, the dominant performance improvement arises from AO-MACE, AWE-wACSF, and AWE-MACE exhibiting broadly comparable accuracy. Notably, the AWE-wACSF model achieves excellent performance despite employing significantly fewer free parameters than the MACE-based architectures, underscoring the importance of physically informed environment encoding rather than model complexity alone.

**Table 1:** Box-Plot Statistics Summarizing the Predictive Performance of the AO–wACSF, AO–MACE, AWE–wACSF, and AWE–MACE Models for XAS and XES When Trained on Element-Specific Data Sets^a^

model	WL_1_	Q1	median	Q3	WH_1_
XAS					
AO–wACSF	0.0004	0.0093	0.0202	0.0398	0.0855
AWE–wACSF	0.0002	0.0085	0.0177	0.0342	0.0727
AO–MACE	0.0004	0.0070	0.0142	0.0272	0.0576
AWE–MACE	0.0001	0.0061	0.0144	0.0312	0.0688
XES					
AO–wACSF	0.0023	0.0341	0.0699	0.1331	0.2814
AWE–wACSF	0.0003	0.0223	0.0545	0.1174	0.2601
AO–MACE	0.0011	0.0245	0.0507	0.0992	0.2110
AWE–MACE	0.0002	0.0137	0.0378	0.0896	0.2029

a The table reports the lower and upper non-outlier bounds (WL_1_, WH_1_) together with the quartiles (Q1, Median, Q3) of the MS–SSIM error distributions shown in Fig. 2, averaged over all transition-metal elements considered.

For XES, the improvement trends are more pronounced, with a clear reduction in error both across the periodic table and along the hierarchy of descriptor sophistication from AO-wACSF to AWE-MACE. This behavior indicates a stronger sensitivity of emission spectra to the enhanced geometric and chemical information captured by equivariant MACE representations, while also revealing that XES benefits more uniformly from inclusion of the environment. As highlighted in Table 1, the AWE-MACE model delivers the best overall performance. Overall, the increased error observed for XES relative to that for XAS reflects the greater sensitivity of emission spectra to subtle variations in the occupied electronic structure. In addition, early 3d transition metals (e.g., Ti and V) exhibit broader chemical diversity within the data set, leading to a more complex structure–spectrum relationship.

Interestingly, and as further corroborated in Tables 1 and S2, the performance of models trained on the combined multielement data set (Table S2) is only marginally inferior to that obtained from element-specific training sets (Table 1). Indeed, for several of the smaller data sets—most notably Cr and V—the jointly trained model exhibits a slight improvement in predictive accuracy. We also note that the AWE models exhibit smaller decreases in predictive accuracy when moving from element-specific to multielement networks. This result indicates that, despite the increased chemical diversity of the training data, the shared learning framework is able to exploit transferable structure–spectrum relationships across different transition-metal environments, thereby partially compensating for the reduced element-specific data density.

To provide deeper insight into model performance, Figure 3 presents box plots of spectral error as a function of descriptor similarity for XAS predictions across the Ti–Zn series. Results are shown for the AO-wACSF (blue), AWE-wACSF (green), AO-MACE (yellow), and AWE-MACE (red) models. Descriptor (either absorber-only wACSF and MACE) similarity is quantified as the distance between the descriptor of a given test structure and its nearest neighbor in the training set, with the absolute value normalized by dividing by the average distance over all training examples. Based on this distance, test examples are partitioned into four regimes, as indicated in Figure 3: *identical* (*d* < 10^–3^), *familiar* (10^–3^ ≤ *d* < 1 × 10^–1.5^), *unfamiliar* (1 × 10^–1.5^ ≤ *d* < 1), and *strange* (*d* ≥ 1).

**Figure 3:**
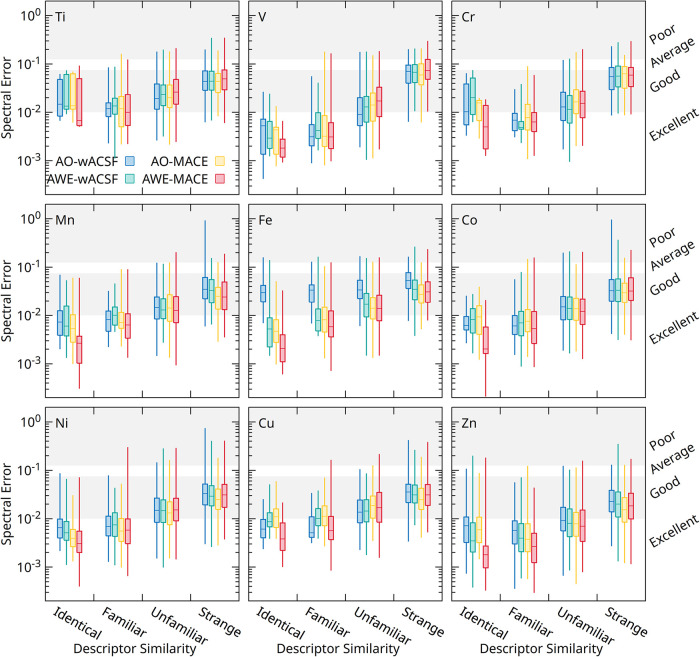
Box plots illustrating the XAS spectral error predicted using the AO-wACSF (blue), AWE-wACSF (green), AO-MACE (yellow), and AWE-MACE (red) models, against the descriptor variance, defined as the distance a test set example input descriptor has from all examples in the training set.

In all cases, a decrease in descriptor similarity—corresponding to test structures lying progressively further from the training distribution—leads to a systematic increase in spectral error. This behavior demonstrates a clear correlation between structural similarity and prediction reliability. Across the full range of descriptor variance, all models exhibit broadly similar error growth with decreasing similarity. This indicates that, while the AWE representations significantly improve overall predictive accuracy, they do not substantially extend the extrapolation capability beyond the training domain. Indeed, in the *strange* regime, the error distributions are remarkably similar across all models, highlighting a common limitation in predicting highly out-of-distribution structures and identifying a clear direction for future methodological development.

A similar trend is observed for the XES predictions, as shown in Figure 4. As in XANES, the spectral error increases systematically with decreasing descriptor similarity across all elements, confirming that extrapolation beyond the training manifold leads to a progressive degradation in predictive accuracy. This effect is particularly pronounced for the late transition metals, where an increased descriptor variance is accompanied by a marked broadening of the error distributions.

**Figure 4:**
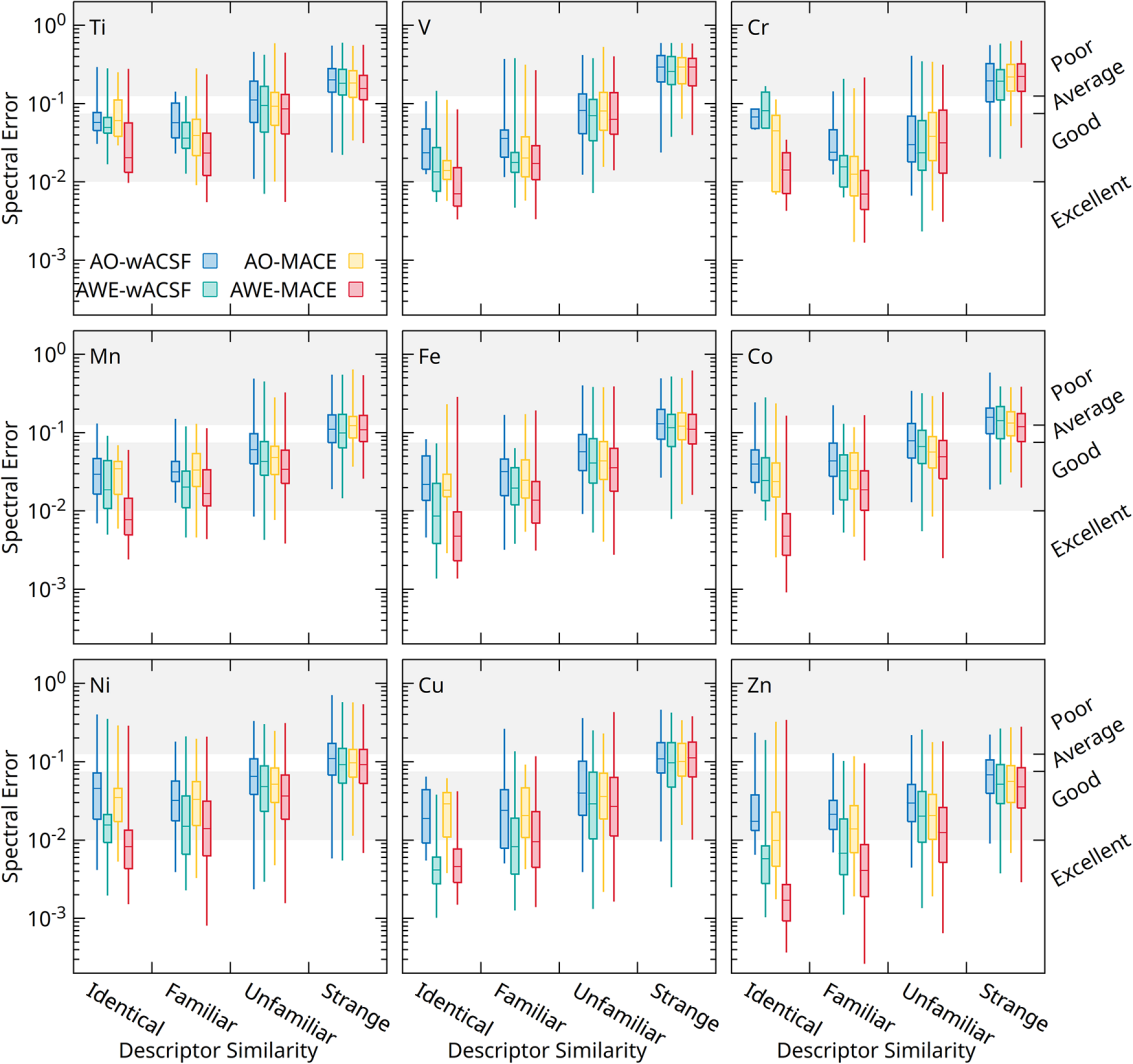
Box plots illustrating the XES spectral error predicted using the AO-wACSF (blue), AWE-wACSF (green), AO-MACE (yellow), and AWE-MACE (red) models, against the descriptor variance, defined as the distance a test set example input descriptor is from all examples in the training set.

Consistent with the XAS results, the AWE-based representations yield lower median errors and tighter interquartile ranges than their AO counterparts, indicating enhanced robustness to structural and chemical diversity. However, in the *strange* regime, the error distributions converge across all models, revealing a shared limitation in predicting highly out-of-distribution structures and reinforcing the conclusion that current improvements primarily enhance interpolation rather than true extrapolation capability.

Further understanding of performance can be obtained by looking directly at the spectra. Figure 5 presents nine representative Ni K-edge XANES spectra predicted using the AO-wACSF (blue), AWE-wACSF (green), AO-MACE (yellow), and AWE-MACE (red) models. The upper three panels show spectra drawn from the 1st–10th percentiles of the error distribution, corresponding to the best-performing predictions. The central three panels display spectra from the 45th–55th percentiles, representative of median performance, while the lower three panels present spectra from the 90th–100th percentiles, corresponding to the worst performers. The top row demonstrates excellent quantitative agreement between all four models and the reference calculated spectra. In the middle row, small deviations begin to emerge, although the predictions remain quantitatively reliable overall, with AO-wACSF (blue) models exhibiting the largest error. The bottom row—corresponding to the lowest-performing cases—exhibits substantial discrepancies, most notably for the **HPNONI** complex, where all models struggle to reproduce key spectral features accurately.

**Figure 5:**
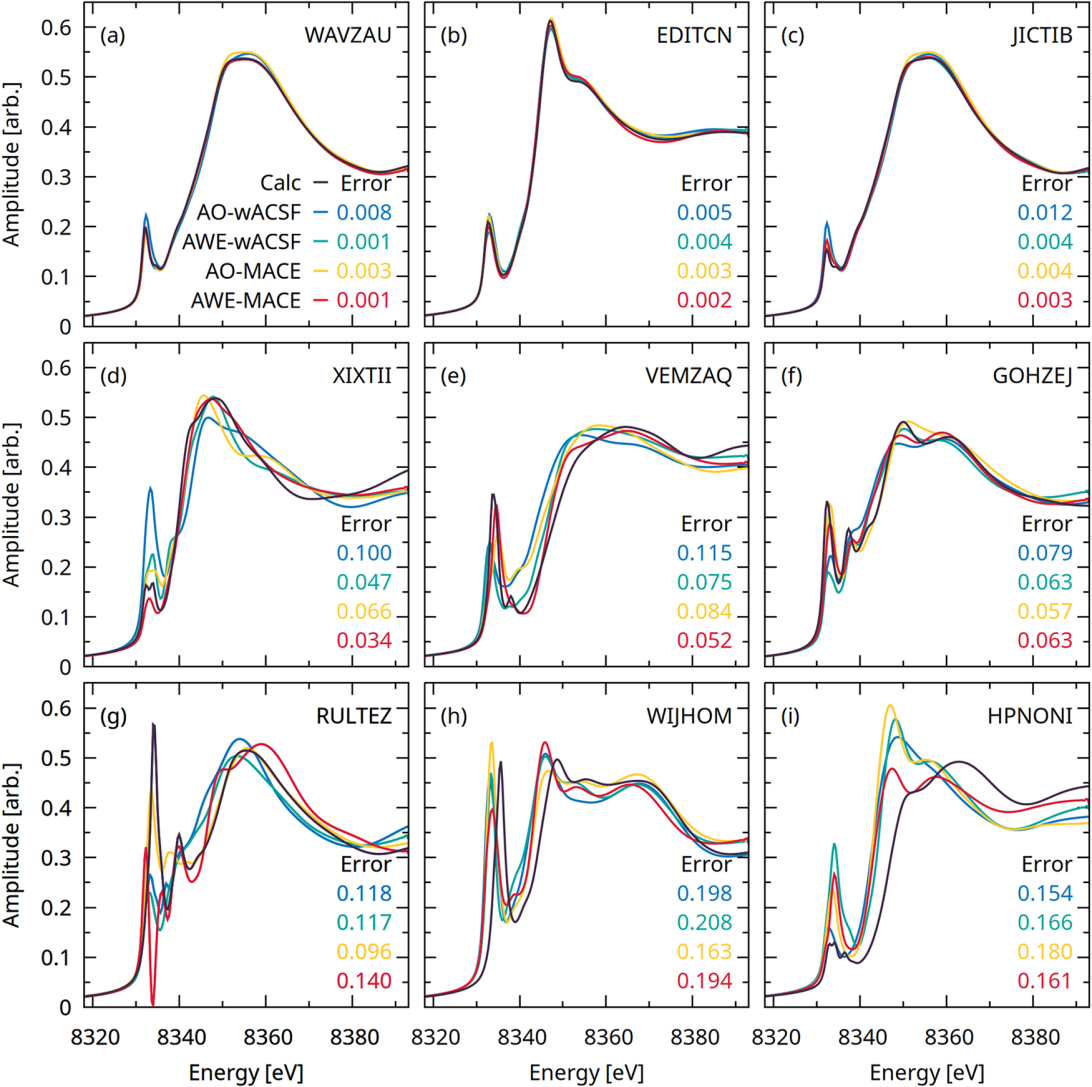
Representative Ni K-edge XANES spectra predicted using the AO-wACSF (blue), AWE-wACSF (green), AO-MACE (yellow), and AWE-MACE (red) models. The upper three panels (a–c) show spectra drawn from the 1st–10th percentiles of the error distribution, corresponding to the best-performing predictions. The central three panels (d–f) display spectra from the 45th–55th percentiles, representative of median performance, while the lower three panels (g–i) present spectra from the 90th–100th percentiles, corresponding to the worst performers. The six-character labels in the lower right corner of each panel denote the Cambridge Structural Database (CSD) reference codes for the corresponding samples. The values reported in each panel are the MS-SSIM errors associated with the respective model predictions.

Figure 6 presents spectra in the same format as Figure 5, but for Ni K-edge XES spectra predicted using the four models introduced in this work. As for XAS, the top row demonstrates excellent quantitative agreement between all four models and the reference calculated XES spectra. In the middle row, small deviations begin to appear—most notably in peak intensities—although the predictions remain quantitatively reliable overall, with the AWE-MACE (red) model exhibiting the best performance. The bottom row, corresponding to the lowest-performing cases, shows more pronounced discrepancies. These are most severe for the **HAYHEV** complex; for the remaining systems, the dominant errors arise from modest peak shifts and variations in relative intensity rather than gross spectral distortions.

**Figure 6:**
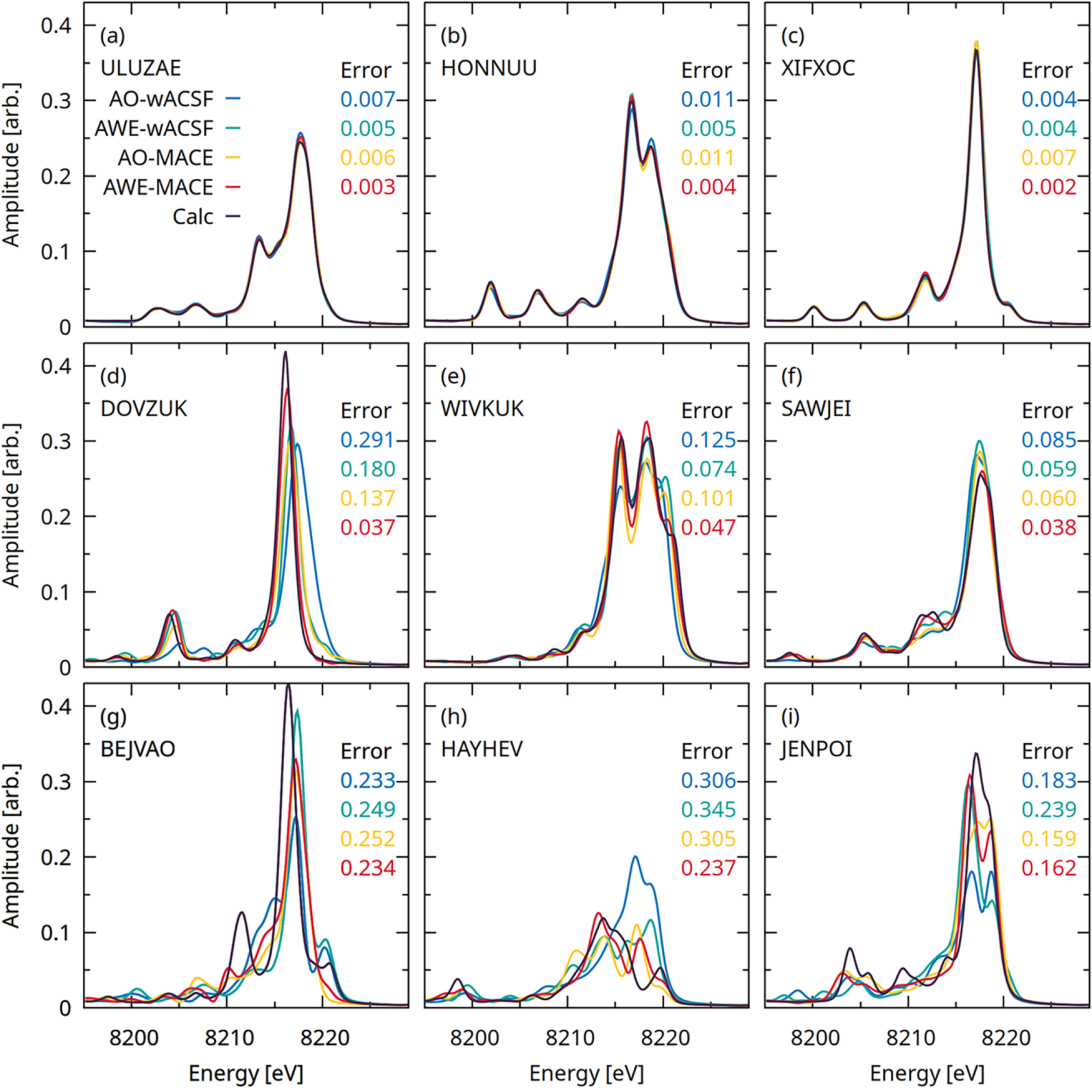
Representative Ni K-edge XES spectra predicted using the AO-wACSF (blue), AWE-wACSF (green), AO-MACE (yellow), and AWE-MACE (red) models. The upper three panels (a–c) show spectra drawn from the 1st–10th percentiles of the error distribution, corresponding to the best-performing predictions. The central three panels (d–f) display spectra from the 45th–55th percentiles, representative of median performance, while the lower three panels (g–i) present spectra from the 90th–100th percentiles, corresponding to the worst performers. The six-character labels in the lower right corner of each panel denote the Cambridge Structural Database (CSD) reference codes for the corresponding samples. The values reported in each panel are the MS-SSIM errors associated with the respective model predictions.

Figures S3–S38 present the corresponding results for all edges, with predictions generated using both atom-specific and all-atom networks. Overall, differences in the XES predictions are marginal, as are the differences in the XAS predictions obtained with the AO-MACE and AWE-MACE models. In contrast, XAS predictions from the all-atom network using AO-wACSF occasionally show a more pronounced degradation in predictive performance. This is reflected in the aggregate errors (Table S1), where the change in error from atom-specific to all-atom models is larger for the AO-wACSF model.

### Uncertainty Quantification

Understanding and accurately assessing the uncertainty arising from predictions is a key piece of information required within the ML workflow if they are to become widely adopted by research communities, especially in supporting nonexpert users to rapidly assess the reliability of their data. Previous work^51,52^ has demonstrated how the bootstrap resampling approach can be used to assess prediction uncertainty, and this approach is applied in this section to assess prediction quality.

Figure 7 presents box plots of the MS-SSIM error for XANES spectra of each element when all transition metals are trained within a single unified model. Here, the difference with Figure 2 is that the training and predictions are performed within the bootstrapping scheme. Also shown is the correlation, for each descriptor set, between the predicted spectral error and the uncertainty estimated via the bootstrap procedure.

**Figure 7:**
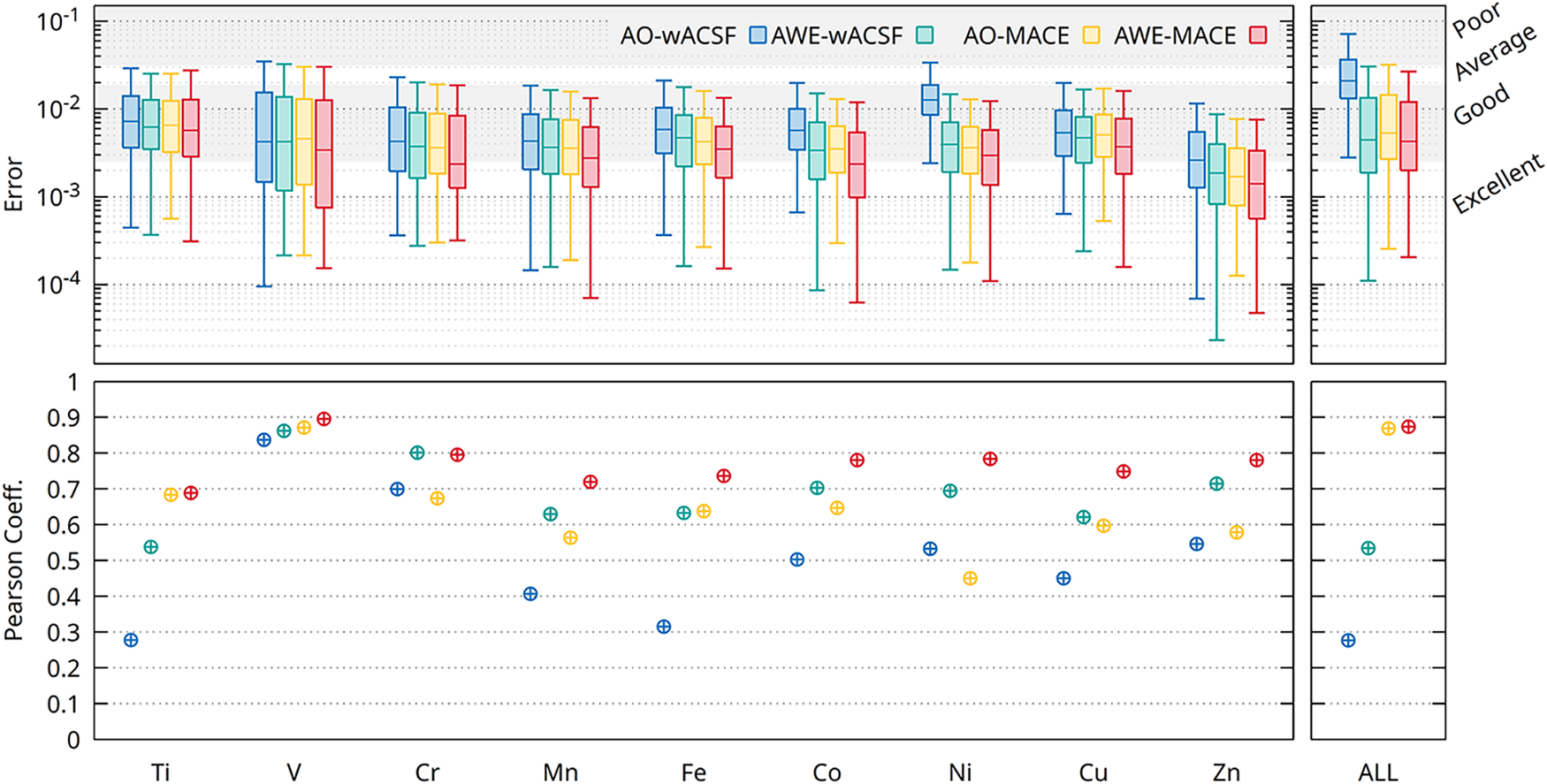
Box plots (upper panel) illustrating the predictive performance of the AO-wACSF (blue), AWE-wACSF (green), AO-MACE (yellow), and AWE-MACE (red) models for XAS across the nine transition-metal K-edges considered in this work, as well as for the model trained on the combined data set (“ALL”). Pearson correlation coefficients (lower panel) between the MS-SSIM error for each spectrum and the predicted standard deviation from the bootstrapped ensembles.

Overall, a moderate-to-strong correlation is observed across all elements, with consistently higher correlations obtained for the AWE-wACSF and AWE-MACE models. This indicates that environment-aware representations provide a more reliable mapping between descriptor space variability and spectral prediction uncertainty. Furthermore, the mean prediction error obtained from the bootstrapped ensembles is systematically lower than that reported in Figure 2, demonstrating that ensemble averaging yields a more accurate and robust description of XAS spectra than individual models alone.

Figure 8 presents the corresponding analysis of the XES spectra. In this case, an even stronger correlation is observed between the MS-SSIM prediction error and the uncertainty estimated from the bootstrapped ensembles across all of the elements. As for XAS, the highest correlations are consistently obtained for the AWE-wACSF and AWE-MACE models, with the AWE-MACE representation exhibiting the most reliable relationship between the predictive accuracy and uncertainty quantification. Moreover, the ensemble models again yield systematically lower prediction errors than the corresponding single-model results, confirming that bootstrapping improves both the accuracy and robustness for XES predictions.

**Figure 8:**
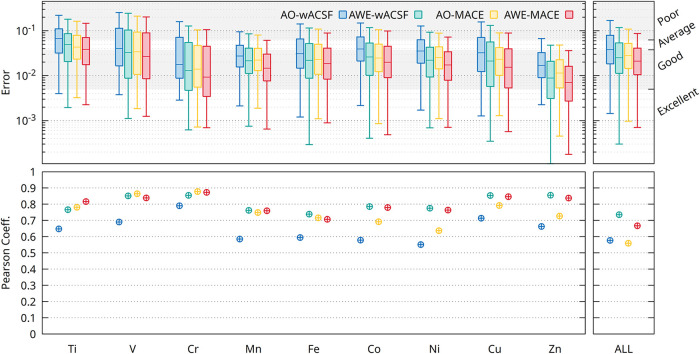
Box plots (upper panel) illustrating the predictive performance of the AO-wACSF (blue), AWE-wACSF (green), AO-MACE (yellow), and AWE-MACE (red) models for XES across the nine transition-metal (TM) K-edges considered in this work, as well as for the model trained simultaneously on the combined data set (“ALL”). Pearson correlation coefficients (lower panel) for each model between MS-SSIM for each spectrum and predicted standard deviation.

### Application of the Model

In the following subsections, we take the models developed above and demonstrate their performance on two specific examples, aimed at demonstrating their sensitivity to compositional variation, their ability to describe the small spectral changes associated with atomic fluctuations, and the transferability of the models between elements.

#### Sensitivity to Compositional Variation at Zn K-Edge

As a first test case, we investigate the ability of our models to reproduce spectral trends arising from systematic compositional variation in a series of Zn complexes: [Zn­(^Me^Im)_4_]^2+^, [Zn­(^Me^Im)_2_(SPh)_2_], [Zn­(SPh)_4_]^2–^, [Zn­(BzO)_2_(SC­(NH_2_)_2_)_2_], and [Zn­(BzO)_2_(pyNH_2_)_2_], as reported in ref 75. In that study, XAS and XES were used to elucidate ligand identity, local coordination geometry, and metal–ligand bond lengths.

Here, our focus is not merely on absolute spectral accuracy, but on whether the neural network can faithfully reproduce observed spectral trends across this chemically diverse ligand series. This constitutes a stringent test of the model’s ability to capture physically meaningful structure–spectrum relationships, rather than simply interpolating within the training distribution.

Figure 9 presents the XAS and XES spectra predicted by using the AO-wACSF and AWE-MACE models. Results for all descriptor sets are reported in Figure S39. The MS-SSIM errors for each complex are shown as insets and are systematically lower for the AWE-MACE model than for AO-wACSF. Beyond improved quantitative accuracy, the AWE-MACE model more faithfully reproduces the relative spectral trends across the ligand series, whereas the AO-wACSF model exhibits noticeable deviations in both the peak positions and intensities. This highlights the importance of environmentally aware, equivariant representations for capturing subtle ligand-dependent electronic structure effects in X-ray spectra.

**Figure 9:**
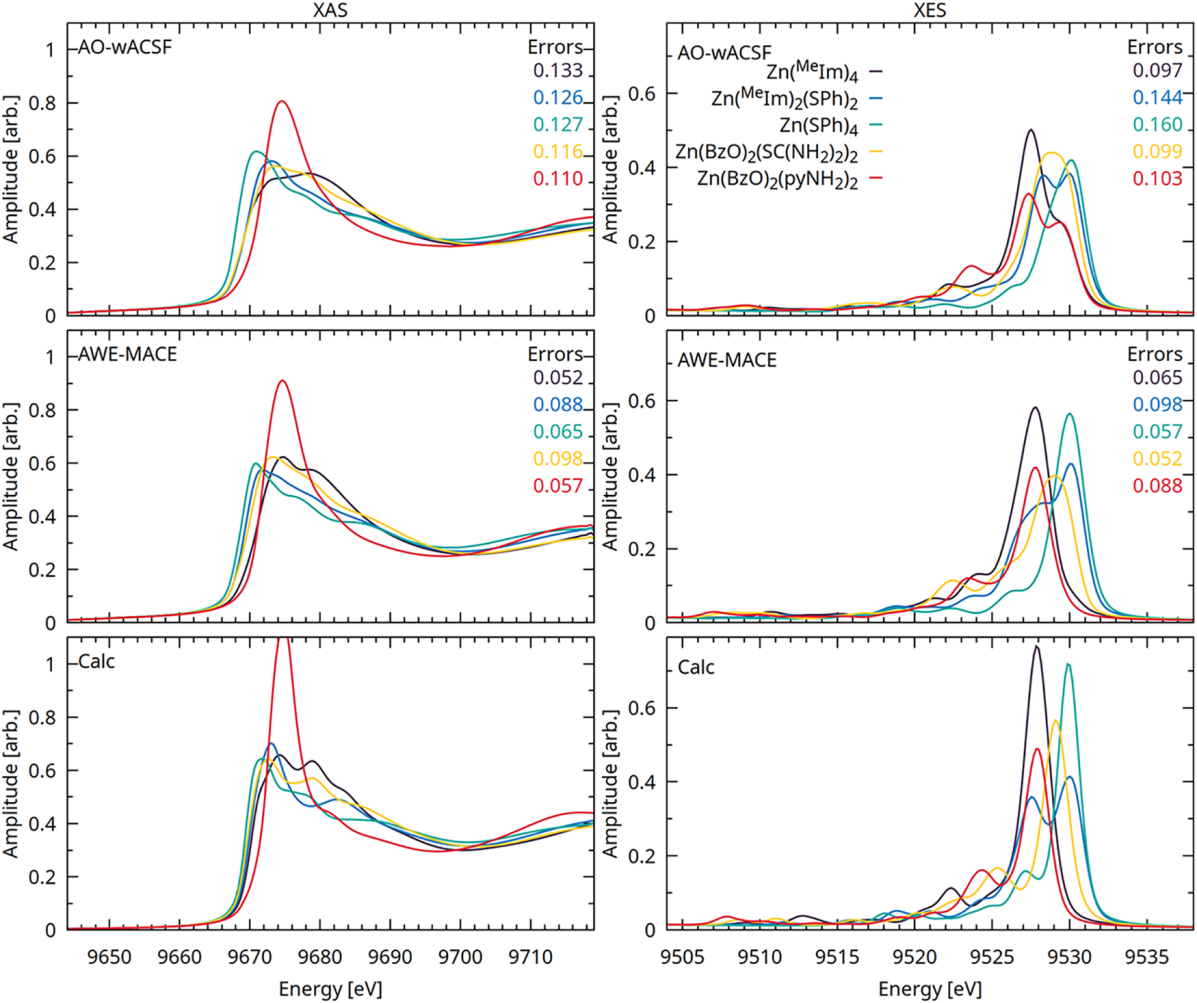
XAS and XES spectra of [Zn­(^Me^Im)_4_]^2+^, [Zn­(^Me^Im)_2_(SPh)_2_], [Zn­(SPh)_4_]^2–^, [Zn­(BzO)_2_(SC­(NH_2_)_2_)_2_], and [Zn­(BzO)_2_(pyNH_2_)_2_]. Spectra are shown for the reference quantum-chemical calculations (using the same level of theory as the training set) and for predictions obtained using the AO-wACSF and AWE-MACE models. Insets report the MS-SSIM errors between the calculated and predicted spectra for each complex.

#### Sensitivity to Geometric Variation at Fe K-Edge

Beyond sensitivity to compositional variation, an equally important benchmark is the ability of the models to capture subtle geometric distortions, such as variations in bond lengths and bond angles. In contrast to ligand substitution, the associated spectral changes are typically much smaller in magnitude, making this a particularly stringent test of the model’s capacity to resolve fine structure–spectrum relationships rather than gross chemical differences.

To probe this regime, we investigate controlled structural perturbations around the Fe center in the porphyrin active site of cytochrome *c*,^76^ which was not part of the training set. A schematic representation of the active site is shown in Figure S40. Specifically, we analyze the evolution of the spectra as a function of systematic variations in (i) the Fe–S distance to the distal methionine ligand, (ii) the Fe–N_His_ distance to the proximal histidine ligand, and (iii) the Fe–N_p_ distances associated with the four pyrrolic nitrogen atoms of the porphyrin macrocycle.

Figure 10 compares the XAS and XES spectra of the doublet ground-state and lowest sextet state structures of cytochrome *c*. The reference quantum-chemical spectra are shown alongside predictions from the AO-wACSF and AWE-MACE models. Overall, the AWE-MACE model exhibits a clear improvement over AO-wACSF, accurately reproducing both the absolute spectral profiles and the differences between the two spin states. The corresponding difference spectra (sextet–doublet), which are directly comparable to the difference experimental spectra reported in ref 76, further highlight this behavior: the AWE-MACE predictions show good agreement with both the calculated and experimental XAS features.

**Figure 10:**
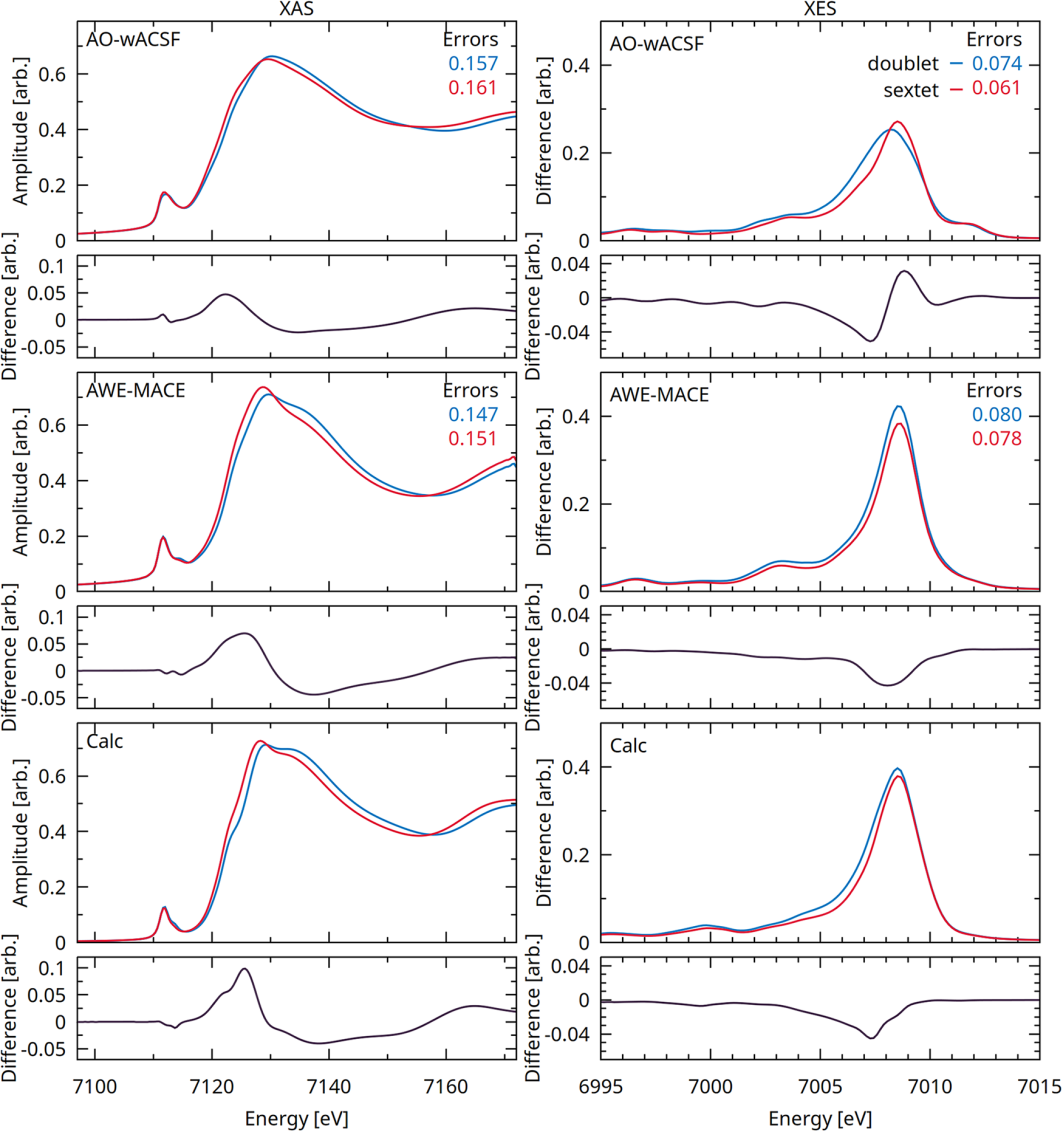
XAS and XES spectra of cytochrome *c* for the doublet ground state and sextet excited state. For each case, the corresponding difference spectrum (Δ = sextet – doublet) is shown beneath the absolute spectra and can be directly compared with the experimental difference spectra reported in ref 76. The highest row is machine-learning predictions from the AO-wACSF, the middle row is predictions using the AWE-MACE models, and the lowest panels present the reference calculated spectra.

Additional insight is provided in Figures S41 and S42, which illustrate the spectral evolution under systematic perturbations of the Fe–S, Fe–N_His_, and Fe–N_*p*_ bond distances. Consistent with the trends observed in Figure 10, the AWE-MACE model most faithfully reproduces the calculated spectral responses to these geometric distortions. Interestingly, despite sulfur being the heaviest ligand in the first coordination sphere, variations in the Fe–S distance exert only a minor influence on both the XAS and XES spectra. This suggests that, for cytochrome *c*, X-ray spectroscopic measurements are intrinsically less sensitive to changes in the Fe–S bond length upon photoexcitation than to distortions within the porphyrin and Fe–N coordination environment.

## Conclusions

In this work, we introduced an absorber-centric, environment-aware extension of the XANESNET framework that explicitly targets two long-standing practical limitations in machine-learned X-ray spectroscopy: the reliance on absorber-only handcrafted structural descriptors and the direct regression of discretized spectral intensities. Our approach augments per-atom representations (either wACSF or equivariant MACE features) with a learnable soft radial environment encoder that aggregates the surrounding chemical neighborhood into a compact latent embedding. In parallel, we represent spectra using a multiscale Gaussian basis with ridge-projected coefficients, providing a smooth, physically structured output space that naturally separates sharp near-edge structure from broader spectral envelopes. Together with a one-dimensional MS-SSIM objective, this yields a unified and data-efficient route from molecular geometry to XAS/XES spectra.

Across the full Ti–Zn benchmark suite, the results demonstrate that explicit environment encoding consistently improves predictive accuracy relative to absorber-only models, with the most pronounced gains observed for XES. While MACE-based descriptors provide a strong baseline, the performance hierarchy observed here shows that the dominant improvement arises from incorporating chemically meaningful environmental information, rather than simply increasing model capacity. Notably, for XAS, the AWE-wACSF model achieves excellent accuracy with substantially fewer parameters, underscoring that the key ingredient is the representation of the local neighborhood rather than architectural complexity alone. Moreover, models trained on a combined multielement data set are only marginally inferior to element-specific models and can even improve performance for the smallest data sets, indicating that transferable structure–spectrum relationships can be learned across chemically diverse transition-metal environments.

We further assessed predictive reliability using bootstrap resampling ensembles, finding moderate-to-strong correlations between the predicted uncertainty (ensemble variance) and the realized MS-SSIM error, with the most reliable uncertainty–error relationships obtained for the environment-aware AWE models. In addition to improving calibration, ensemble averaging systematically reduces prediction error for both XAS and XES, reinforcing bootstrapping as a practical and scalable strategy for uncertainty quantification in structure-to-spectrum workflows. At the same time, the descriptor-similarity analysis highlights a shared limitation across all architectures: performance degrades systematically as test structures move away from the training manifold, and the error distributions converge in highly out-of-distribution regimes. This indicates that the present advances primarily enhance interpolation robustness, while genuine extrapolative capability remains an open challenge.

Finally, we demonstrated the practical value of the framework on chemically and structurally controlled case studies. For the Zn ligand series, the environment-aware AWE-MACE model not only reduces absolute errors but also more faithfully reproduces ligand-dependent spectral trends, consistent with its improved encoding of subtle metal–ligand electronic structure effects. For the Fe porphyrin active-site distortions, the same methodology provides a stringent test of sensitivity to small geometric perturbations, a regime where predictive models must capture fine structure–spectrum relationships rather than large chemical shifts. Looking forward, the combination of learnable environment encoders, physically structured spectral bases, and uncertainty-aware inference provides a natural foundation for extending machine-learned X-ray spectroscopy toward broader chemical coverage, active-learning data set expansion, and more reliable deployment in experimental interpretation pipelines.

## Supplementary Material



## Notes

Supporting results on the influence of the spectral representation, the loss function, and the performance of the bootstrapping simulations (PDF)
