# Supplementary material for: From Local Atomic Structure to X‑ray Spectra: Absorber-Centric Machine-Learning Encoding
Source: J Phys Chem A. 2026 May 6;130(20):3981–95. doi: 10.1021/acs.jpca.6c01127 (PMC13200179; doi:10.1021/acs.jpca.6c01127)
Supplement: Supplementary file 1 [file jp6c01127_si_001.pdf]

Supporting Information: From Local Atomic Structure to X-ray Spectra: Absorber-Centric Machine-Learning Encoding

Thomas James Pope,[†] Bowen Li,[‡] Hendrik Junkawitsch,[¶,§] Annika Bande,[¶,‖] and Thomas James Penfold[*,†]

†*Chemistry – School of Natural and Environmental Sciences, Newcastle University, Newcastle upon Tyne, NE1 7RU, UK*

‡*Research Software Engineer Group, Newcastle University, Newcastle upon Tyne, NE1 7RU, UK*

¶*Helmholtz-Zentrum Berlin für Materialien und Energie GmbH, Theory of Electron Dynamics and Spectroscopy, Hahn-Meitner-Platz 1, 14109 Berlin*

§*Institute for Computer Science, Humboldt-Universität zu Berlin, Unter den Linden 6, 10099 Berlin*

‖*Leibniz University Hannover Institut of Inorganic Chemistry, Callinstr. 9, 30167 Hannover*

E-mail: tom.penfold@newcastle.ac.uk

Dataset sizes

Table S1: Summary of the number of samples, N_{samples}, in each of the nine first-row transition metal {Ti, V, Cr, Mn, Fe, Co, Ni, Cu, Zn} reference datasets for XAS and XES.

Edge	$N_{\text{XASsamples}}$	$N_{\text{XESsamples}}$
Ti	2752	2637
V	1107	1096
Cr	1417	1388
Mn	1694	1650
Fe	4329	4229
Co	3184	2943
Ni	8655	8208
Cu	2804	2510
Zn	5772	5510
All	31714	30171

Analysis of MS-SSIM Error Metric

Figures S1 and S2 show a comparison of the MS-SSIM and MSE error metrics for nine Ni XAS and XES spectra, respectively. To compare the error metrics, each has been normalized by dividing by the average error for the test data set. In each case, the black line shows the calculated spectrum, the blue line the presented spectrum, the yellow line the normalized MSE at each point, and the red line the normalized MS-SSIM error at each point.

Overall inspection of the metrics and the trend support the observation that the MS-SSIM loss is a more appropriate metric than MSE. Indeed, the MSE profiles (orange curves) exhibit sharp, highly localized spikes, particularly in regions of steep spectral gradients such as pre-edge features and intense peaks. These spikes arise because the MSE penalizes pointwise amplitude differences quadratically, making it extremely sensitive to small energy shifts or minor discrepancies in peak height. Consequently, spectra that are visually and physically very similar to the reference can nevertheless yield comparatively large MSE contributions concentrated at a few energy points.

In contrast, the MS-SSIM profiles (red curves) are distributed more broadly across the spectrum and correlate more closely with perceptible differences in spectral shape. MS-SSIM evaluates local correlations in intensity, contrast, and structure over an energy window, and therefore assigns relatively low error to spectra that preserve the correct line shape and relative feature ordering, even when small energy shifts are present. This behaviour is evident, for example, in molecules where the predicted spectra capture the correct multi-peak structure but exhibit slight energy misalignment: the MSE highlights narrow error spikes, whereas SSIM reflects the overall structural agreement between predicted and reference spectra. Importantly, the cases with the largest SSIM errors correspond to qualitatively incorrect spectral predictions, such as missing peaks, incorrect relative intensities, or distorted band shapes. By contrast, large MSE values often arise from minor peak shifts that do not substantially alter the physical interpretation of the spectrum.

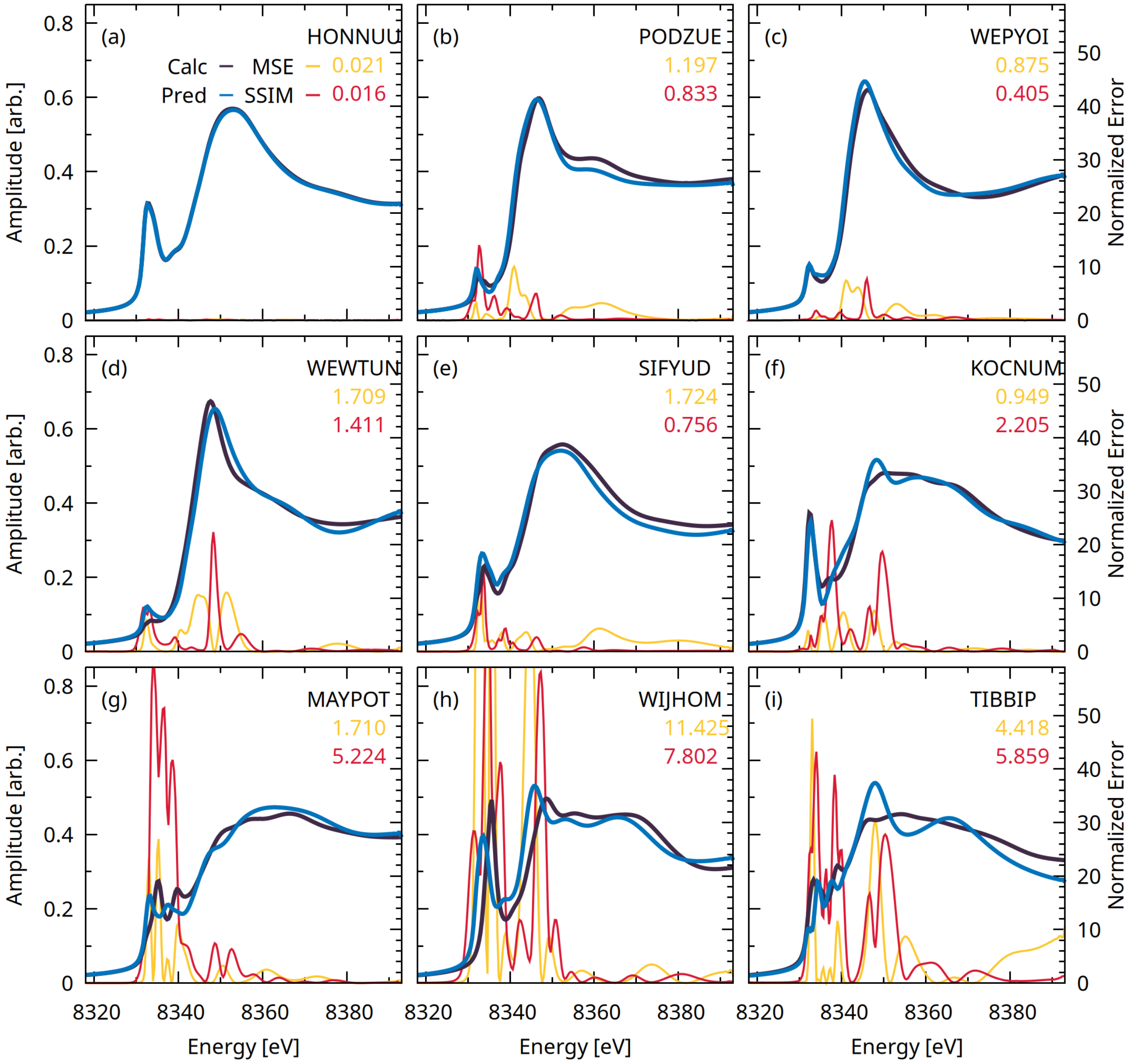

Figure S1: Comparison of the error metrics MS-SSIM and MSE for nine Ni XAS spectra. The MS-SSIM error increases progressively from panels (a) to (i). In each panel, the reference calculated spectrum is shown in black and the predicted spectrum in blue. The pointwise MS-SSIM contribution is plotted in red, while the corresponding MSE contribution is shown in yellow. The six-character labels in the lower-right corner of each panel indicate the Cambridge Structural Database (CSD) reference codes for the corresponding complexes.

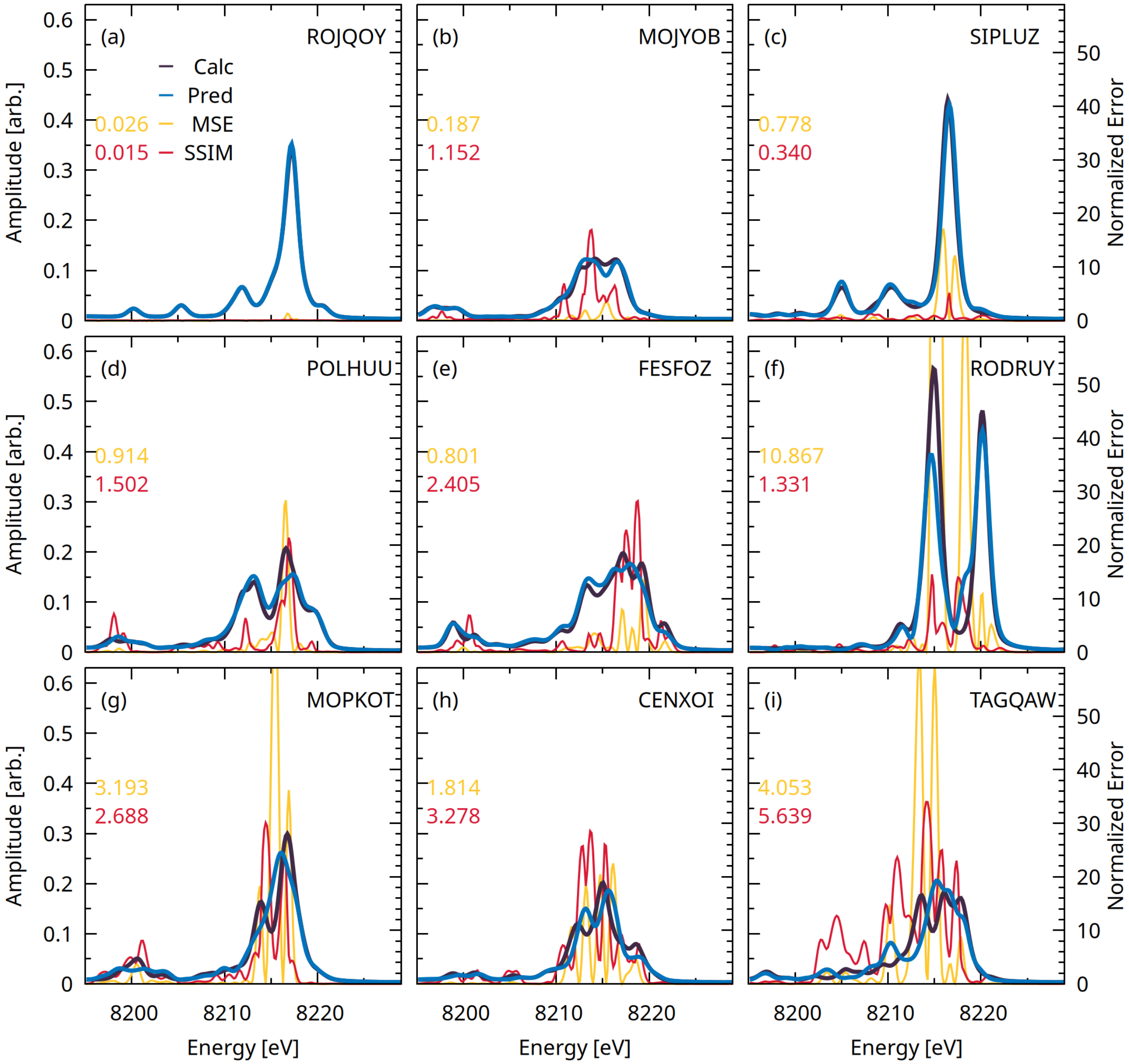

Figure S2: Comparison of the error metrics MS-SSIM and MSE for nine Ni valence-to-core XES spectra. The MS-SSIM error increases progressively from panels (a) to (i). In each panel, the reference calculated spectrum is shown in black and the predicted spectrum in blue. The pointwise MS-SSIM contribution is plotted in red, while the corresponding MSE contribution is shown in yellow. The six-character labels in the lower-right corner of each panel indicate the Cambridge Structural Database (CSD) reference codes for the corresponding complexes.

Supplementary Results

Summary of Performance for "ALL" model

Table S2: Box-plot statistics summarizing the predictive performance of the AO–wACSF, AO–MACE, AWE–wACSF, and AWE–MACE models for XAS and XES when trained on the All-atom datasets. The table reports the lower and upper non-outlier bounds (WL_1, WH_1) together with the quartiles (Q1, Q2, Q3) of the MS–SSIM error distributions. In brackets, we include the percentage error change compared to the atom-specific networks.

**Model**	**WL_1**	**Q1**	**Q2 (Median)**	**Q3**	**WH_1**
**XAS**					
AO–wACSF	0.0009(+153)	0.0156(+68)	0.0306(+51)	0.0782(+96)	0.1721(+101)
AWE–wACSF	0.0006(+287)	0.0090(+5)	0.0199(+12)	0.0571(+67)	0.1290(+77)
AO–MACE	0.0008(+78)	0.0105(+50)	0.0206(+45)	0.0537(+97)	0.1185(+106)
AWE–MACE	0.0009(+875)	0.0096(+58)	0.0200(+39)	0.0561(+80)	0.1253(+82)
**XES**					
AO-wACSF	0.0029(+23)	0.0433(+27)	0.0870(+24)	0.1637(+23)	0.3441(+22)
AWE-wACSF	0.0010(+263)	0.0303(+36)	0.0632(+16)	0.1268(+8)	0.2714(+4)
AO-MACE	0.0017(+54)	0.0346(+41)	0.0649(+28)	0.1157(+17)	0.2373(+12)
AWE-MACE	0.0006(+206)	0.0178(+30)	0.0407(+8)	0.0881(-2)	0.1928(-5)

Comparing the "ALL"-atom and element-specific training regimes shows that element-resolved datasets generally yield improved predictive performance. However, particularly for XES, the differences between the two approaches—quantified by the percentage error changes reported in brackets—are relatively modest, indicating a notable degree of transferability across different absorbing elements. Importantly, the relative ordering of the models remains unchanged: environment-aware (AWE) variants consistently outperform their absorber-only (AO) counterparts, and MACE-based descriptors achieve the lowest overall errors. Nevertheless, the absolute performance gains associated with explicitly encoding the local atomic environment are more clearly resolved in the element-specific setting. This suggests that element-resolved training reduces cross-element variability inherent in the "ALL"-atom models, thereby allowing the advantages of the underlying descriptors and model architectures to be more fully realised.

Influence of Gaussian Basis Representation

Table S3: Box-plot statistics illustrating the influence of the spectral representation (discretised energy points, Fourier transform (FT), and Gaussian basis functions (GBF)) on the performance of AO–MACE model for XAS and XES when trained on element-specific datasets. The table reports the lower and upper non-outlier bounds (WL_1, WH_1) together with the quartiles (Q1, Q2, Q3) of the MS–SSIM error distributions, averaged over all transition-metal elements considered.

Model	$\mathbf{WL_1}$	**Q1**	**Q2 (Median)**	**Q3**	$\mathbf{WH_1}$
**XAS**					
Energy	0.00044	0.00702	0.01422	0.02715	0.05758
FT	0.00208	0.00950	0.01759	0.03243	0.06680
Energy & FT	0.00291	0.01007	0.01780	0.03314	0.06768
GBF, {0.5, 1.0, 2.0, 4.0}, stride: 2	0.00163	0.02012	0.05175	0.10169	0.22254
GBF, {0.5, 1.0, 2.0, 4.0}, stride: 4	0.00433	0.03934	0.06420	0.10113	0.19294
GBF, {0.5, 1.0, 2.0}, stride: 2	0.00082	0.00834	0.01620	0.03039	0.06345
GBF, {1.0, 2.0}, stride: 2	0.00294	0.01262	0.02379	0.04982	0.10558
GBF, {1.0, 2.0}, stride: 4	0.00134	0.01140	0.01888	0.03209	0.06307
**XES**					
Energy	0.00203	0.03425	0.06987	0.13263	0.28008
FFT	0.00396	0.03782	0.07977	0.15020	0.31876
Energy & FFT	0.00410	0.03662	0.07831	0.15028	0.32066
GBF, {0.5, 1.0, 2.0, 4.0}, stride: 2	0.00315	0.04217	0.08129	0.14389	0.29612
GBF, {0.5, 1.0, 2.0, 4.0}, stride: 4	0.00272	0.03930	0.07739	0.13880	0.28791
GBF, {0.5, 1.0, 2.0}, stride: 2	0.00427	0.04483	0.08541	0.14608	0.29754
GBF, {1.0, 2.0}, stride: 2	0.00372	0.03955	0.07929	0.13973	0.28971
GBF, {1.0, 2.0}, stride: 4	0.00317	0.03616	0.07339	0.13662	0.28667

Spectral Plots of Performance for Held-Out Examples

XAS

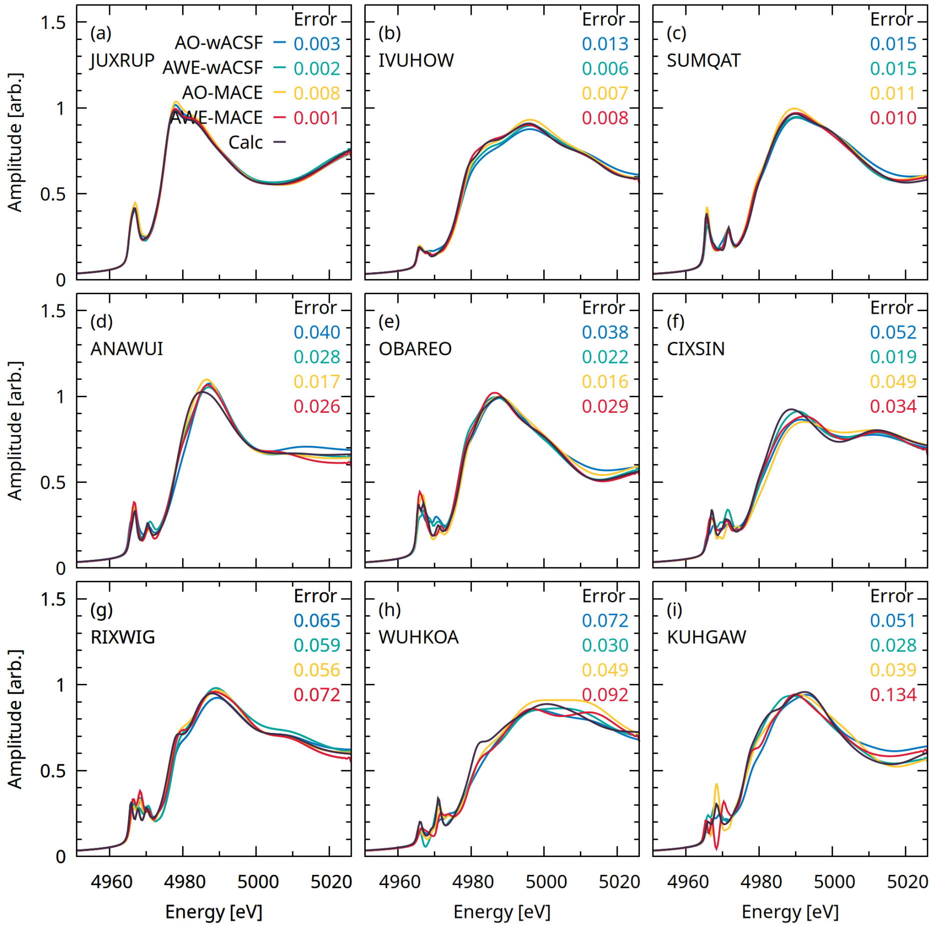

Figure S3: Representative Ti K-edge XANES spectra predicted using the AO-wACSF (blue), AWE-wACSF (green), AO-MACE (yellow), and AWE-MACE (red) models. The upper three panels show spectra drawn from the 1st–15th percentiles of the error distribution, corresponding to the best-performing predictions. The central three panels display spectra from the 45th–55th percentiles, representative of median performance, while the lower three panels present spectra from the 85th–100th percentiles, corresponding to the worst performers. The six-character labels in the lower right corner of each panel denote the Cambridge Structural Database (CSD) reference codes for the corresponding samples. The values reported in each panel are the MS-SSIM errors associated with the respective model predictions.

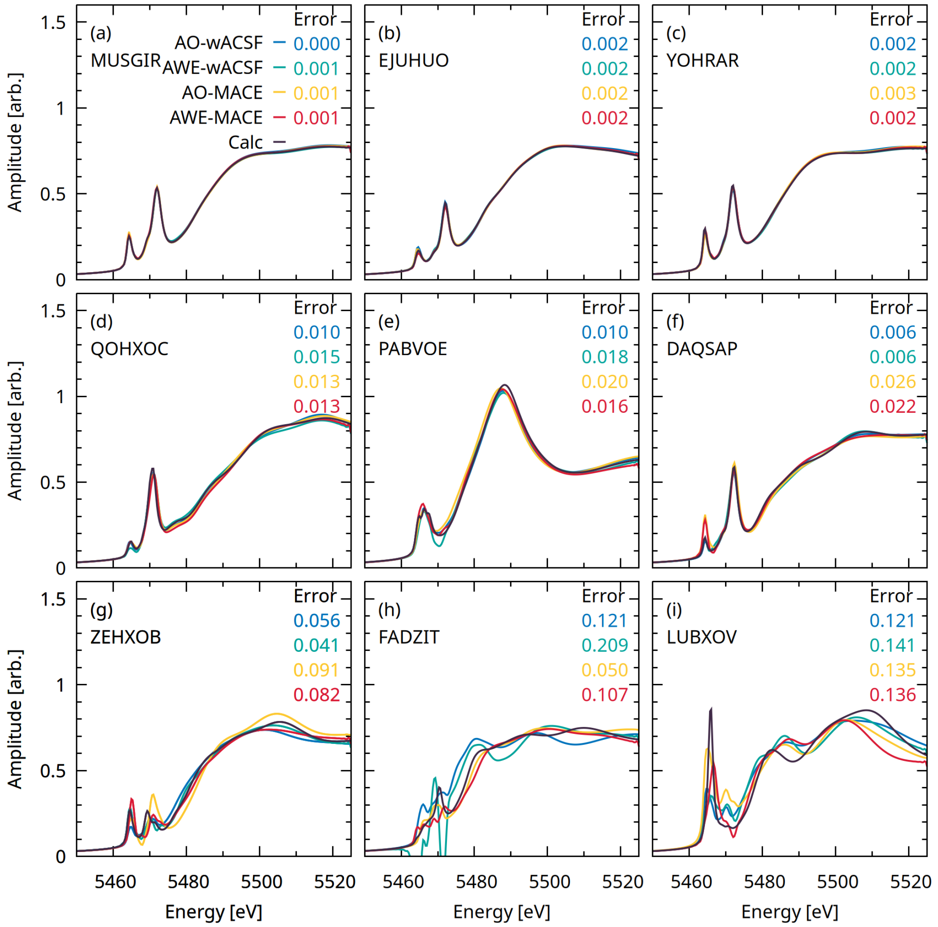

Figure S4: Representative V K-edge XANES spectra predicted using the AO-wACSF (blue), AWE-wACSF (green), AO-MACE (yellow), and AWE-MACE (red) models. The upper three panels show spectra drawn from the 1st–15th percentiles of the error distribution, corresponding to the best-performing predictions. The central three panels display spectra from the 45th–55th percentiles, representative of median performance, while the lower three panels present spectra from the 85th–100th percentiles, corresponding to the worst performers. The six-character labels in the lower right corner of each panel denote the Cambridge Structural Database (CSD) reference codes for the corresponding samples. The values reported in each panel are the MS-SSIM errors associated with the respective model predictions.

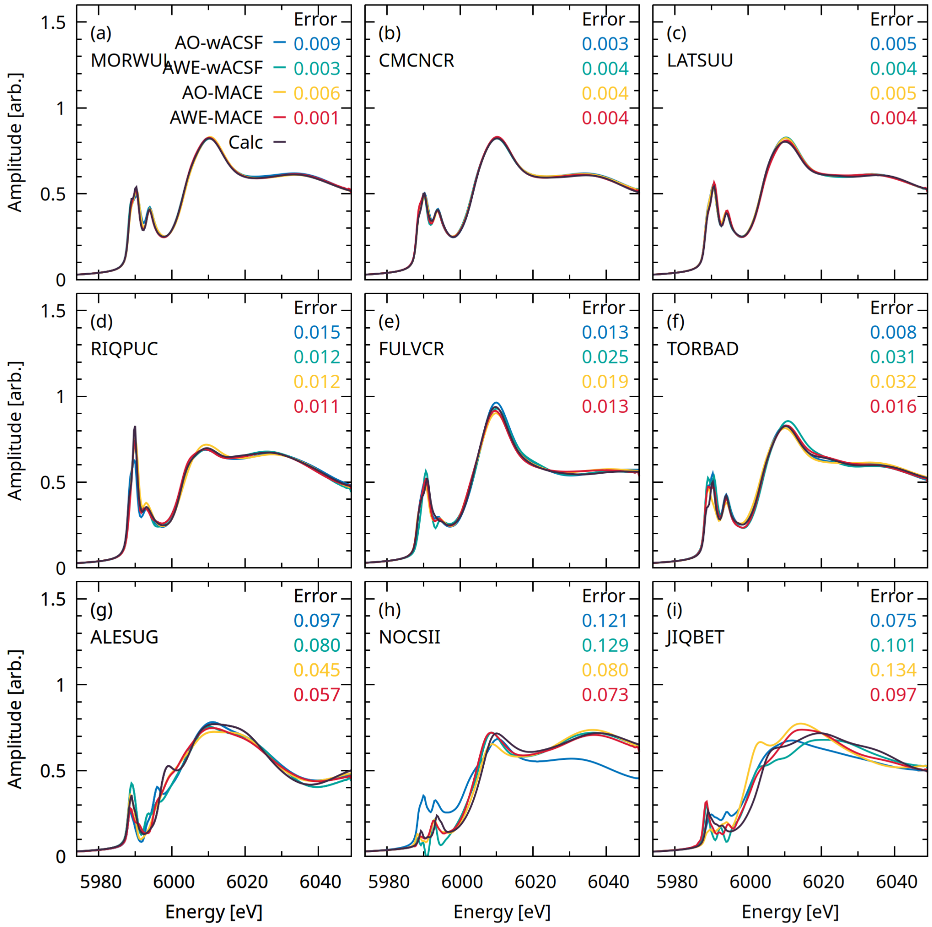

Figure S5: Representative Cr K-edge XANES spectra predicted using the AO-wACSF (blue), AWE-wACSF (green), AO-MACE (yellow), and AWE-MACE (red) models. The upper three panels show spectra drawn from the 1st–15th percentiles of the error distribution, corresponding to the best-performing predictions. The central three panels display spectra from the 45th–55th percentiles, representative of median performance, while the lower three panels present spectra from the 85th–100th percentiles, corresponding to the worst performers. The six-character labels in the lower right corner of each panel denote the Cambridge Structural Database (CSD) reference codes for the corresponding samples. The values reported in each panel are the MS-SSIM errors associated with the respective model predictions.

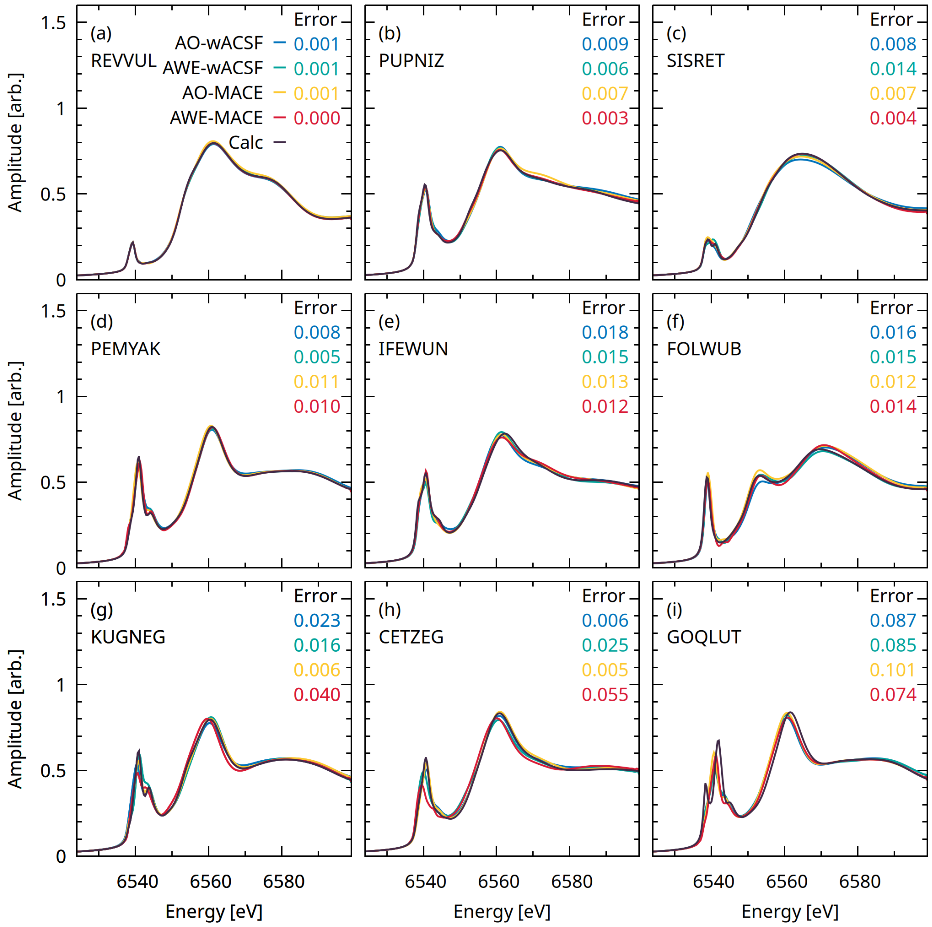

Figure S6: Representative Mn K-edge XANES spectra predicted using the AO-wACSF (blue), AWE-wACSF (green), AO-MACE (yellow), and AWE-MACE (red) models. The upper three panels show spectra drawn from the 1st–15th percentiles of the error distribution, corresponding to the best-performing predictions. The central three panels display spectra from the 45th–55th percentiles, representative of median performance, while the lower three panels present spectra from the 85th–100th percentiles, corresponding to the worst performers. The six-character labels in the lower right corner of each panel denote the Cambridge Structural Database (CSD) reference codes for the corresponding samples. The values reported in each panel are the MS-SSIM errors associated with the respective model predictions.

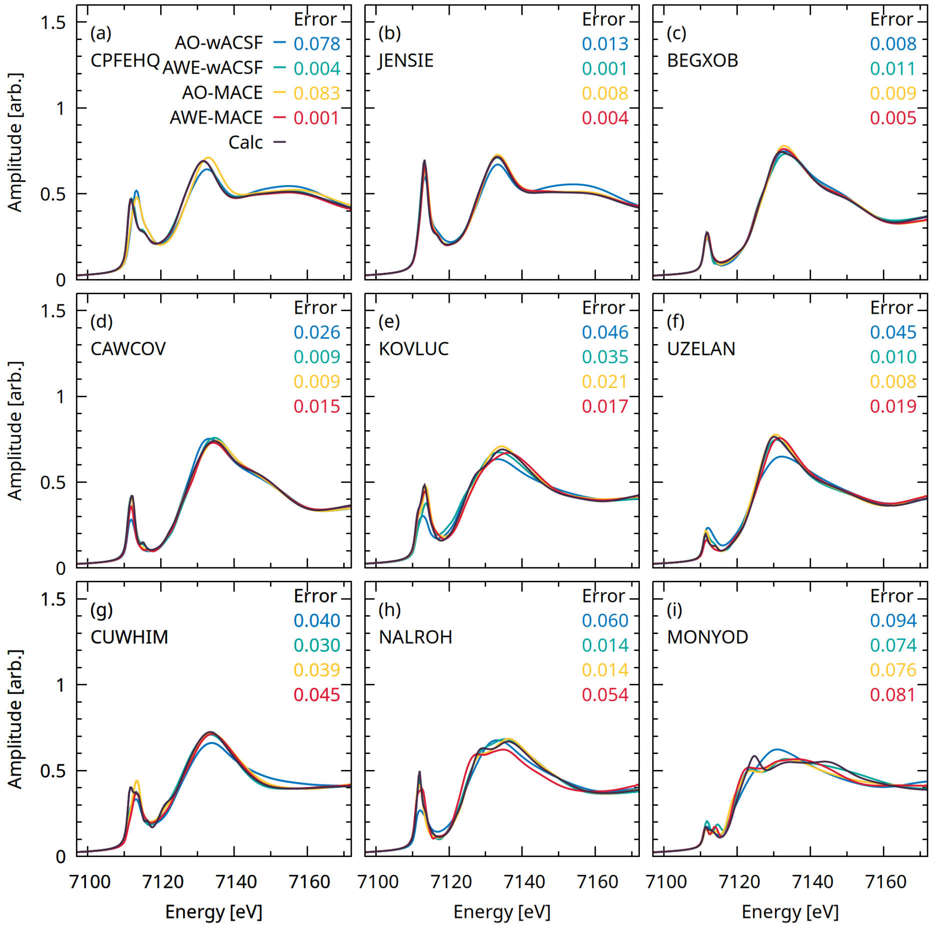

Figure S7: Representative Fe K-edge XANES spectra predicted using the AO-wACSF (blue), AWE-wACSF (green), AO-MACE (yellow), and AWE-MACE (red) models. The upper three panels show spectra drawn from the 1st–15th percentiles of the error distribution, corresponding to the best-performing predictions. The central three panels display spectra from the 45th–55th percentiles, representative of median performance, while the lower three panels present spectra from the 85th–100th percentiles, corresponding to the worst performers. The six-character labels in the lower right corner of each panel denote the Cambridge Structural Database (CSD) reference codes for the corresponding samples. The values reported in each panel are the MS-SSIM errors associated with the respective model predictions.

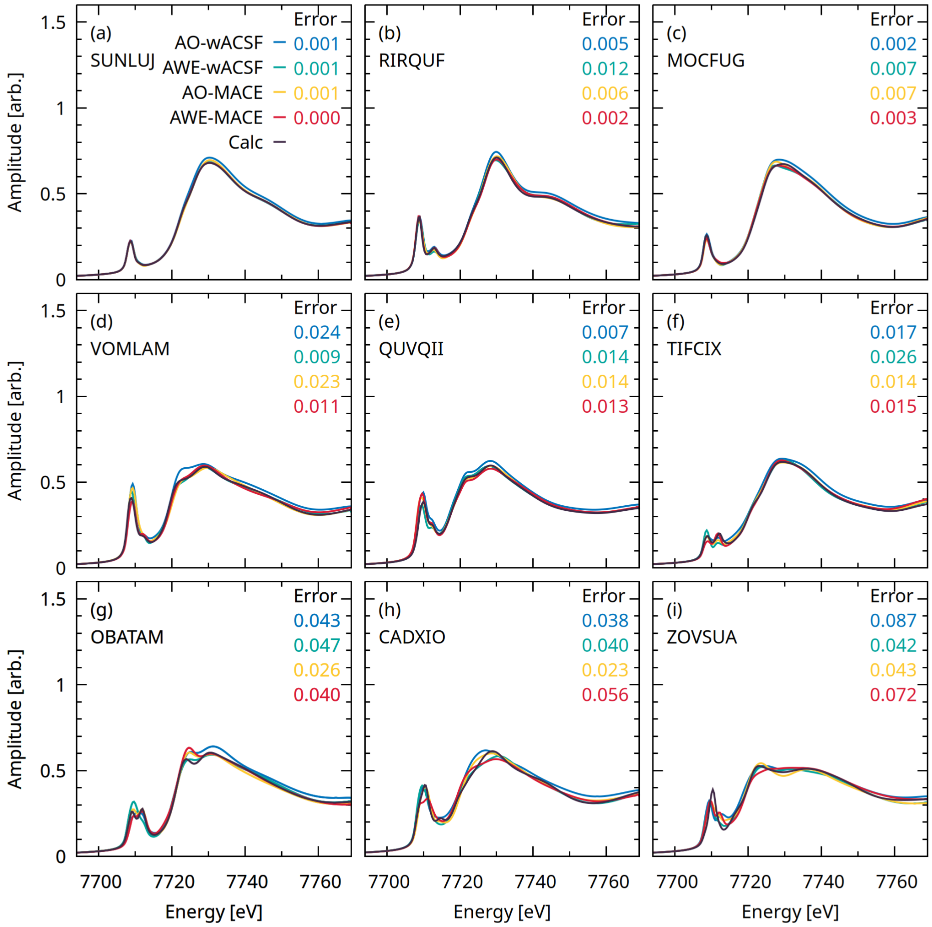

Figure S8: Representative Co K-edge XANES spectra predicted using the AO-wACSF (blue), AWE-wACSF (green), AO-MACE (yellow), and AWE-MACE (red) models. The upper three panels show spectra drawn from the 1st–15th percentiles of the error distribution, corresponding to the best-performing predictions. The central three panels display spectra from the 45th–55th percentiles, representative of median performance, while the lower three panels present spectra from the 85th–100th percentiles, corresponding to the worst performers. The six-character labels in the lower right corner of each panel denote the Cambridge Structural Database (CSD) reference codes for the corresponding samples. The values reported in each panel are the MS-SSIM errors associated with the respective model predictions.

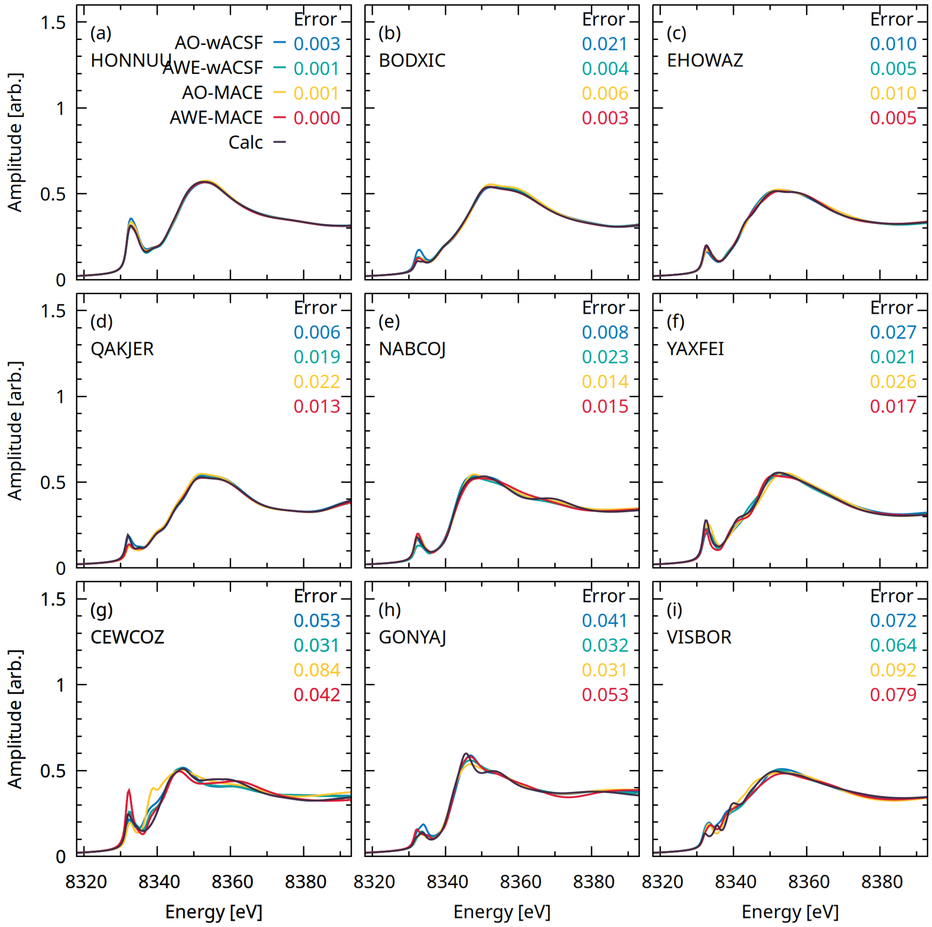

Figure S9: Representative Ni K-edge XANES spectra predicted using the AO-wACSF (blue), AWE-wACSF (green), AO-MACE (yellow), and AWE-MACE (red) models. The upper three panels show spectra drawn from the 1st–15th percentiles of the error distribution, corresponding to the best-performing predictions. The central three panels display spectra from the 45th–55th percentiles, representative of median performance, while the lower three panels present spectra from the 85th–100th percentiles, corresponding to the worst performers. The six-character labels in the lower right corner of each panel denote the Cambridge Structural Database (CSD) reference codes for the corresponding samples. The values reported in each panel are the MS-SSIM errors associated with the respective model predictions.

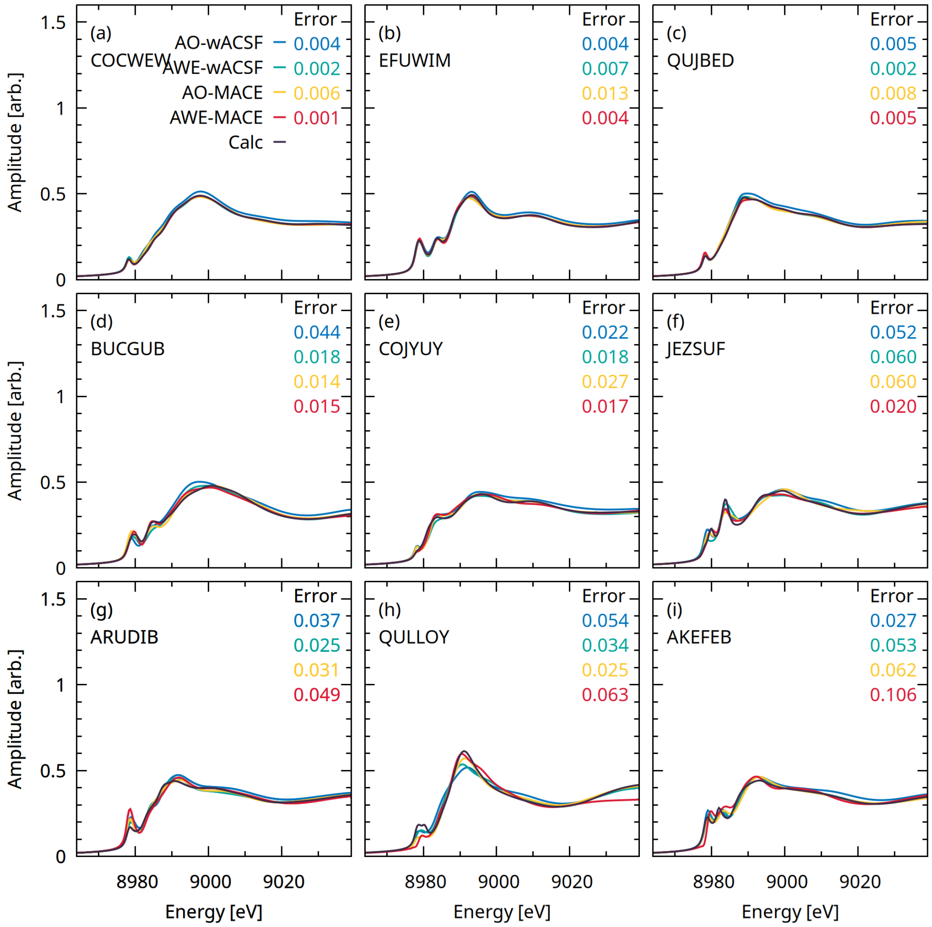

Figure S10: Representative Cu K-edge XANES spectra predicted using the AO-wACSF (blue), AWE-wACSF (green), AO-MACE (yellow), and AWE-MACE (red) models. The upper three panels show spectra drawn from the 1st–15th percentiles of the error distribution, corresponding to the best-performing predictions. The central three panels display spectra from the 45th–55th percentiles, representative of median performance, while the lower three panels present spectra from the 85th–100th percentiles, corresponding to the worst performers. The six-character labels in the lower right corner of each panel denote the Cambridge Structural Database (CSD) reference codes for the corresponding samples. The values reported in each panel are the MS-SSIM errors associated with the respective model predictions.

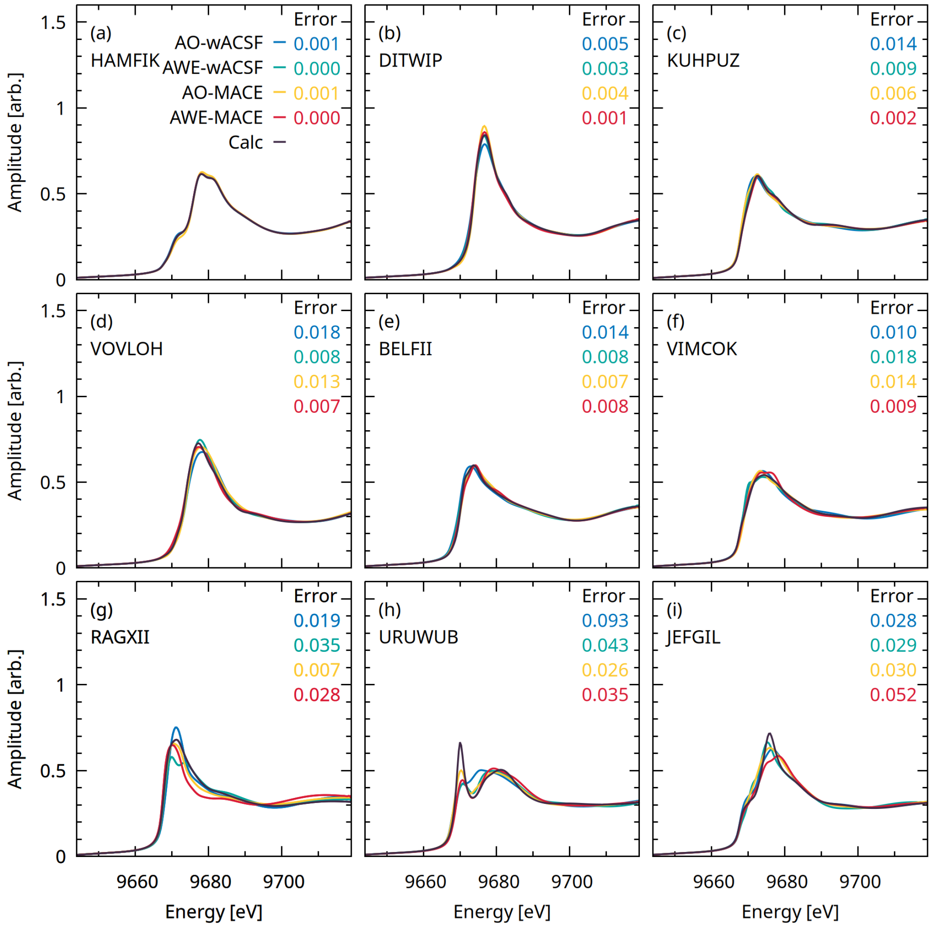

Figure S11: Representative Zn K-edge XANES spectra predicted using the AO-wACSF (blue), AWE-wACSF (green), AO-MACE (yellow), and AWE-MACE (red) models. The upper three panels show spectra drawn from the 1st–15th percentiles of the error distribution, corresponding to the best-performing predictions. The central three panels display spectra from the 45th–55th percentiles, representative of median performance, while the lower three panels present spectra from the 85th–100th percentiles, corresponding to the worst performers. The six-character labels in the lower right corner of each panel denote the Cambridge Structural Database (CSD) reference codes for the corresponding samples. The values reported in each panel are the MS-SSIM errors associated with the respective model predictions.

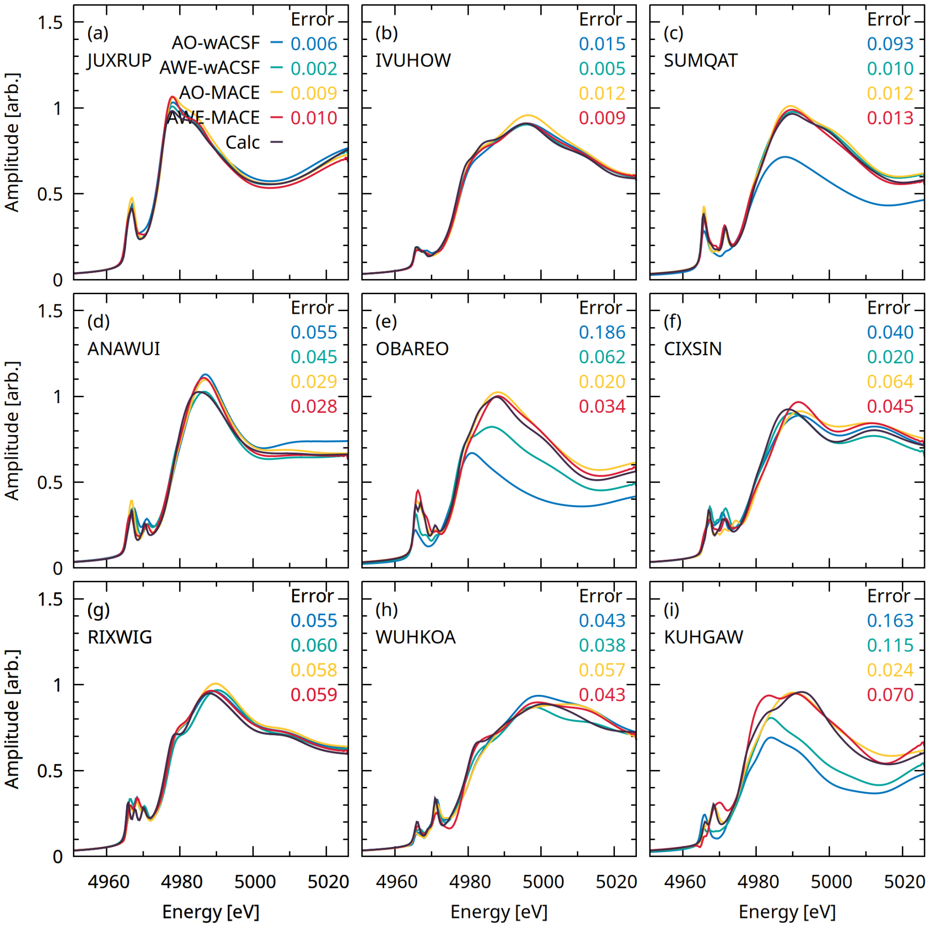

Figure S12: Equivalent to Fig. S3, showing XAS predictions obtained using a model trained on the combined "ALL"-atom dataset rather than element-specific data.

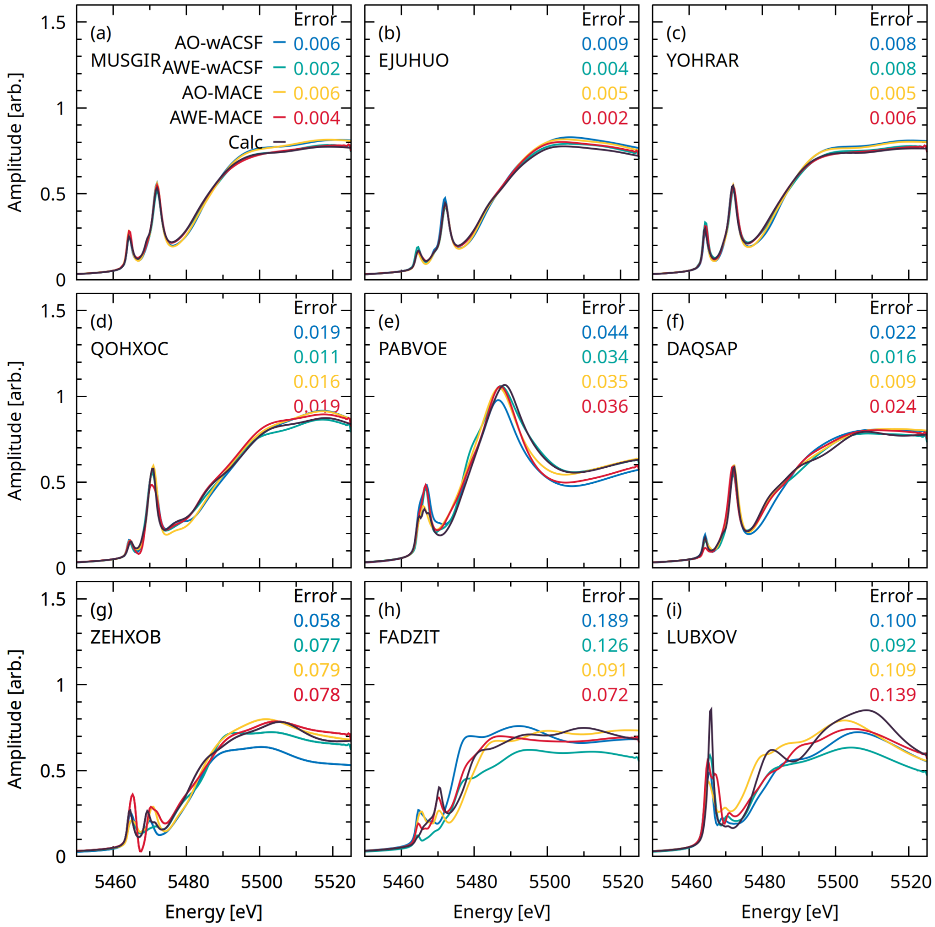

Figure S13: Equivalent to Fig. S4, showing XAS predictions obtained using a model trained on the combined "ALL"-atom dataset rather than element-specific data.

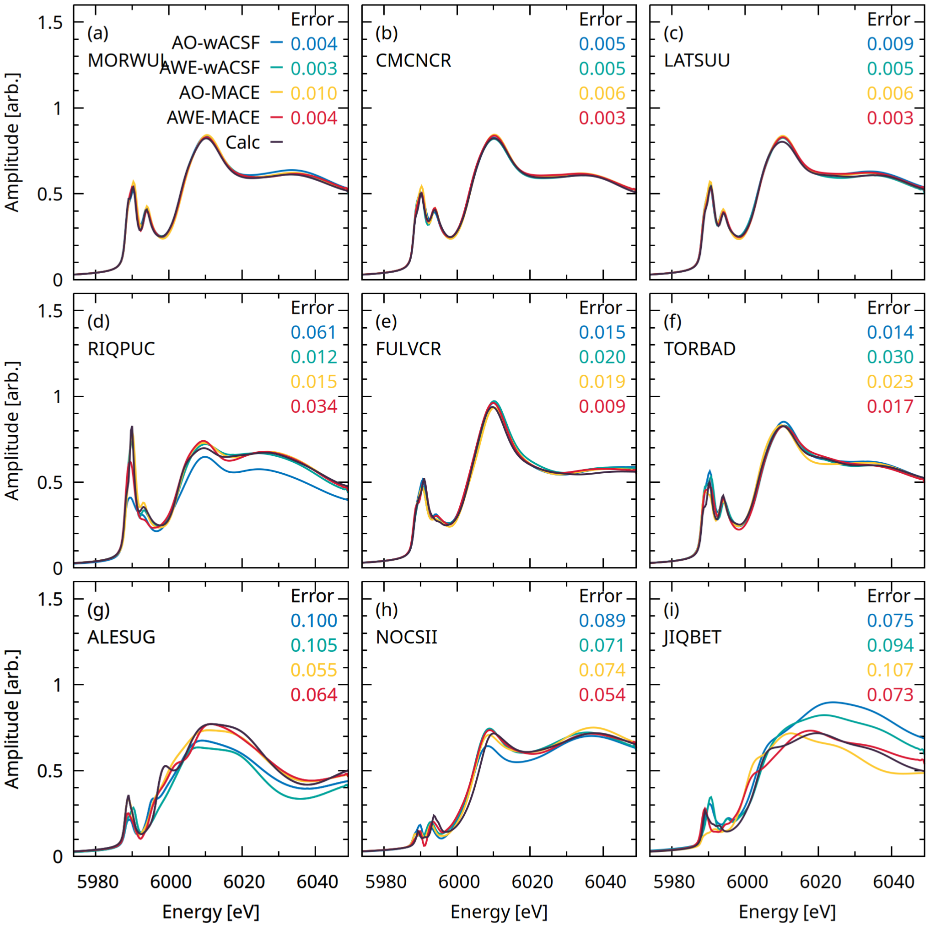

Figure S14: Equivalent plot to S5, showing XAS predictions obtained using a model trained on the combined “ALL”-atom dataset rather than element-specific data.

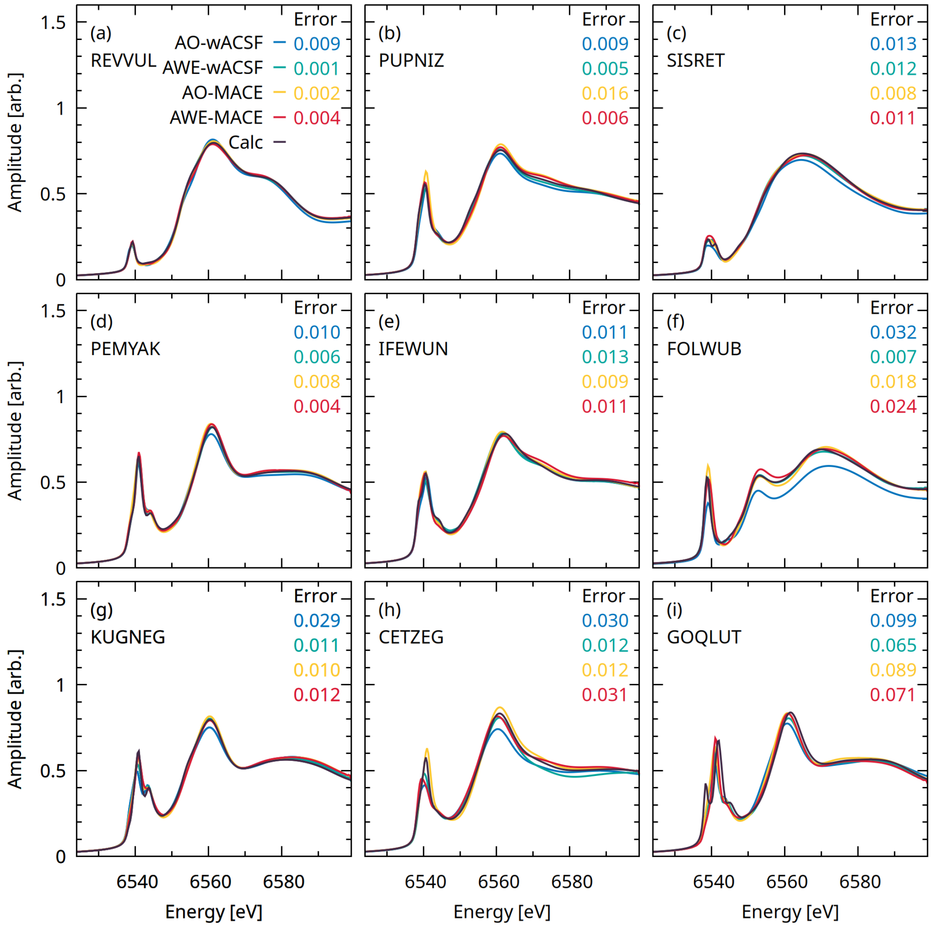

Figure S15: Equivalent plot to S6, showing XAS predictions obtained using a model trained on the combined "ALL"-atom dataset rather than element-specific data.

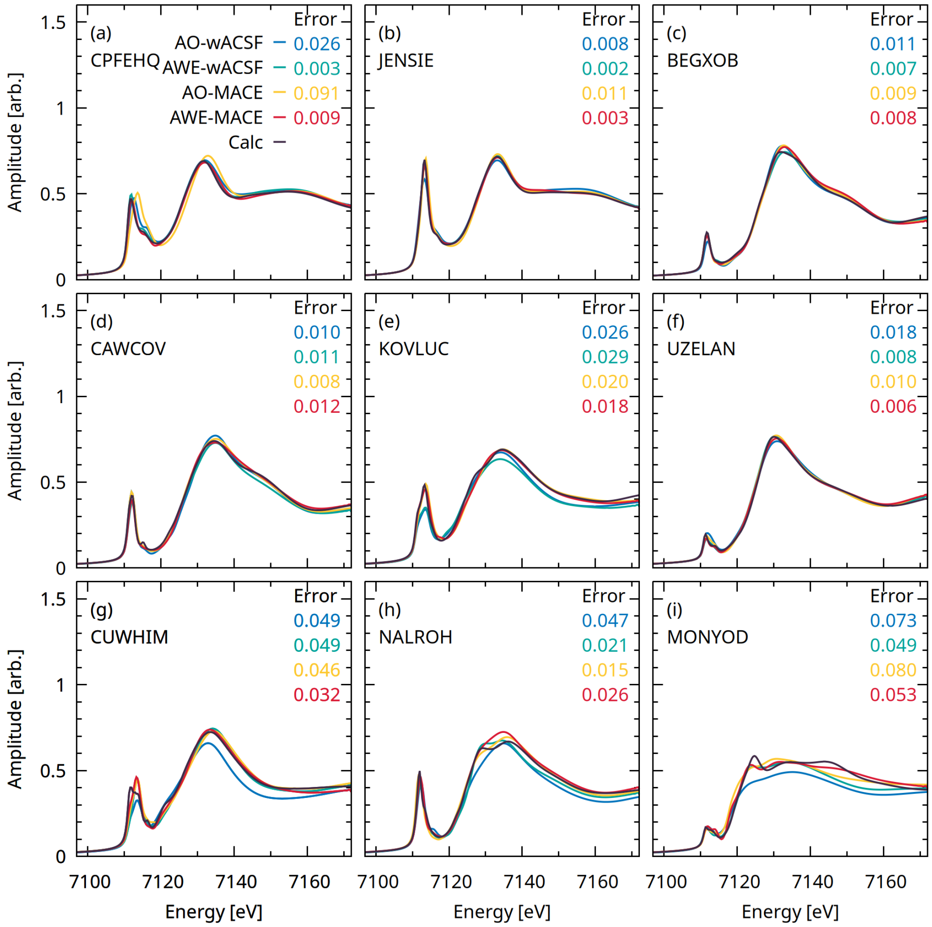

Figure S16: Equivalent plot to S7, showing XAS predictions obtained using a model trained on the combined "ALL"-atom dataset rather than element-specific data.

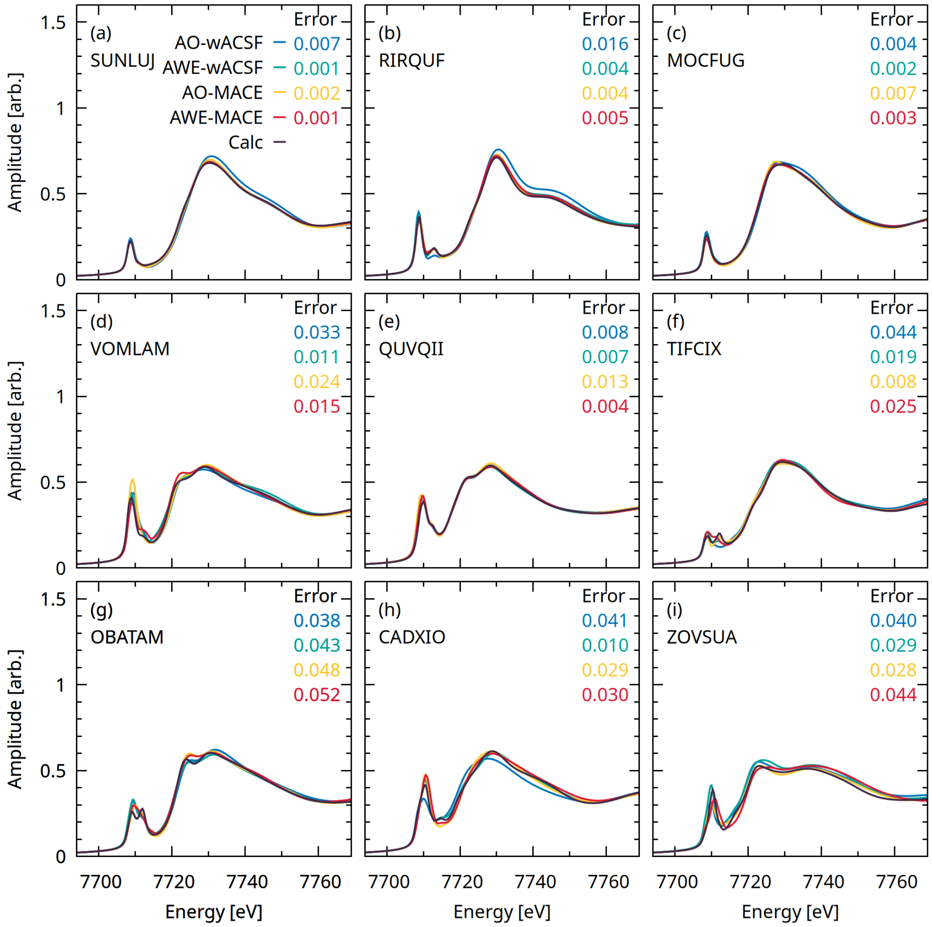

Figure S17: Equivalent plot to S8, showing XAS predictions obtained using a model trained on the combined "ALL"-atom dataset rather than element-specific data.

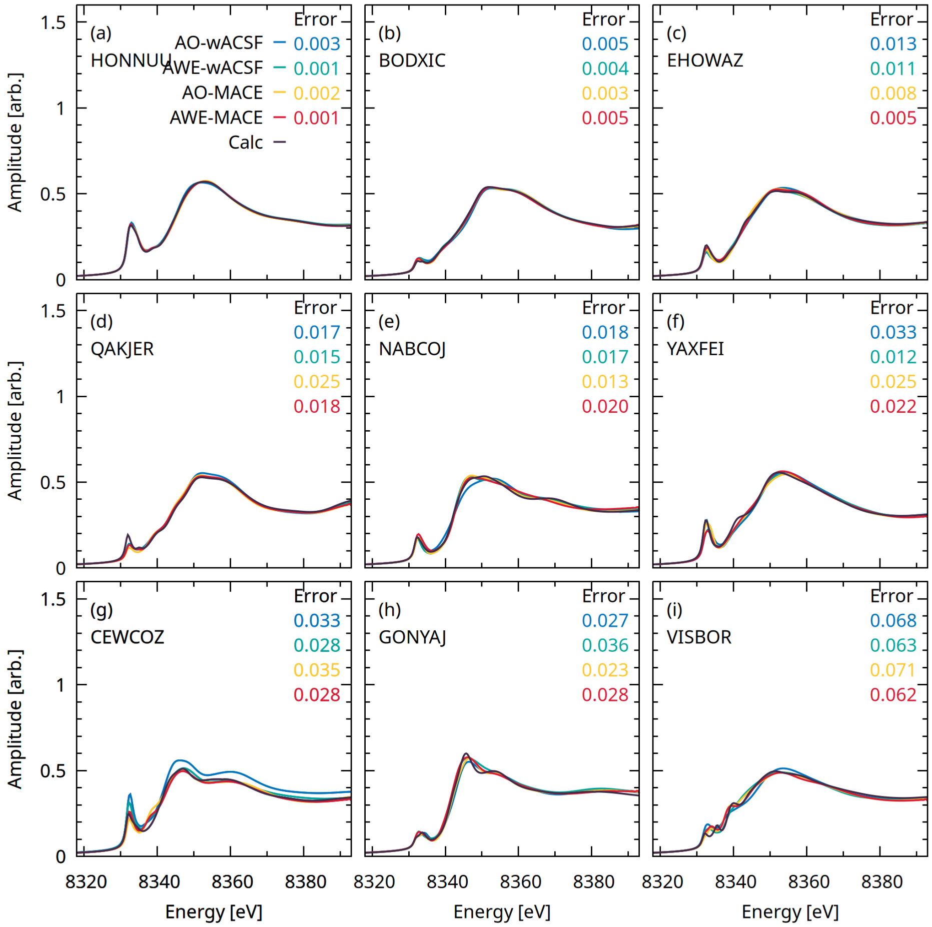

Figure S18: Equivalent plot to S9, showing XAS predictions obtained using a model trained on the combined "ALL"-atom dataset rather than element-specific data.

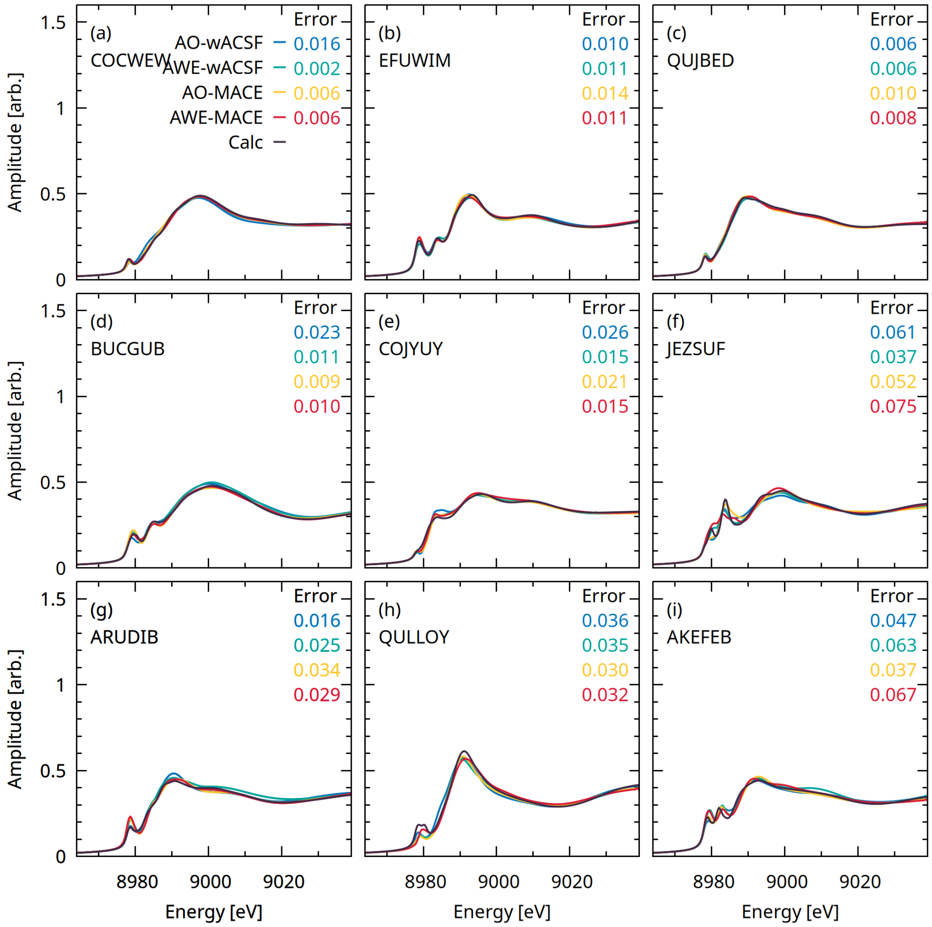

Figure S19: Equivalent plot to S10, showing XAS predictions obtained using a model trained on the combined "ALL"-atom dataset rather than element-specific data.

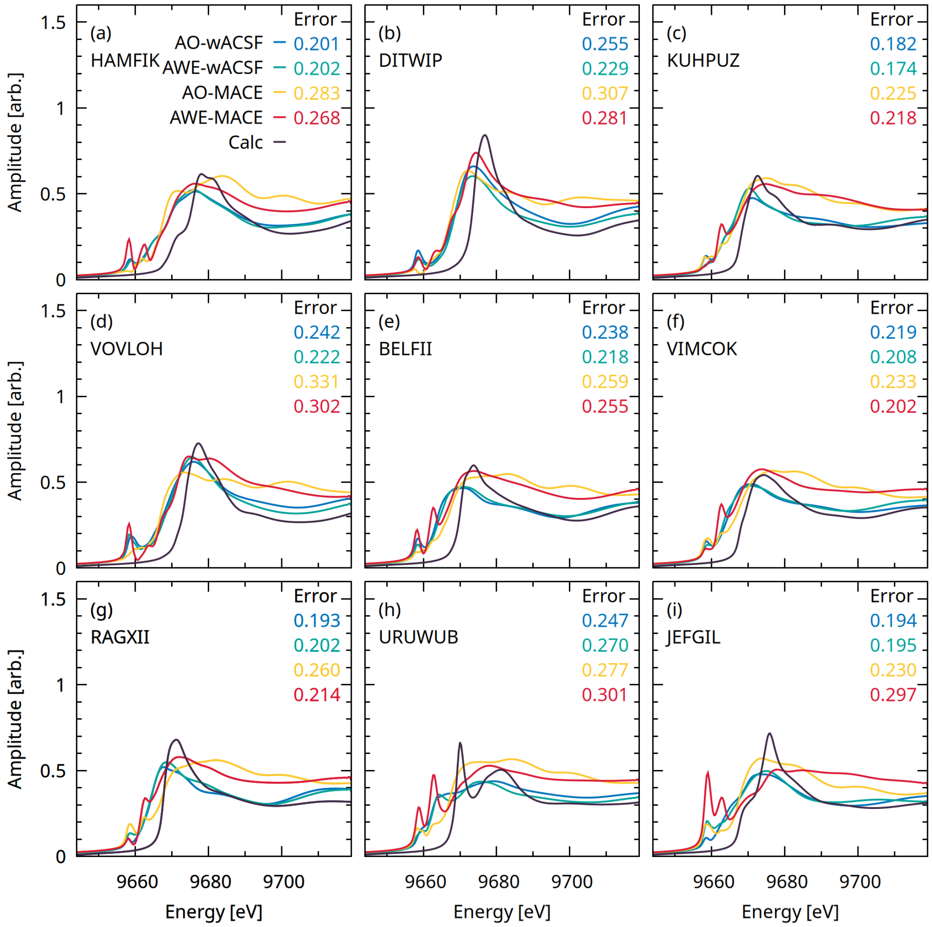

Figure S20: Equivalent plot to S11, showing XAS predictions obtained using a model trained on the combined “ALL”-atom dataset rather than element-specific data.

XES

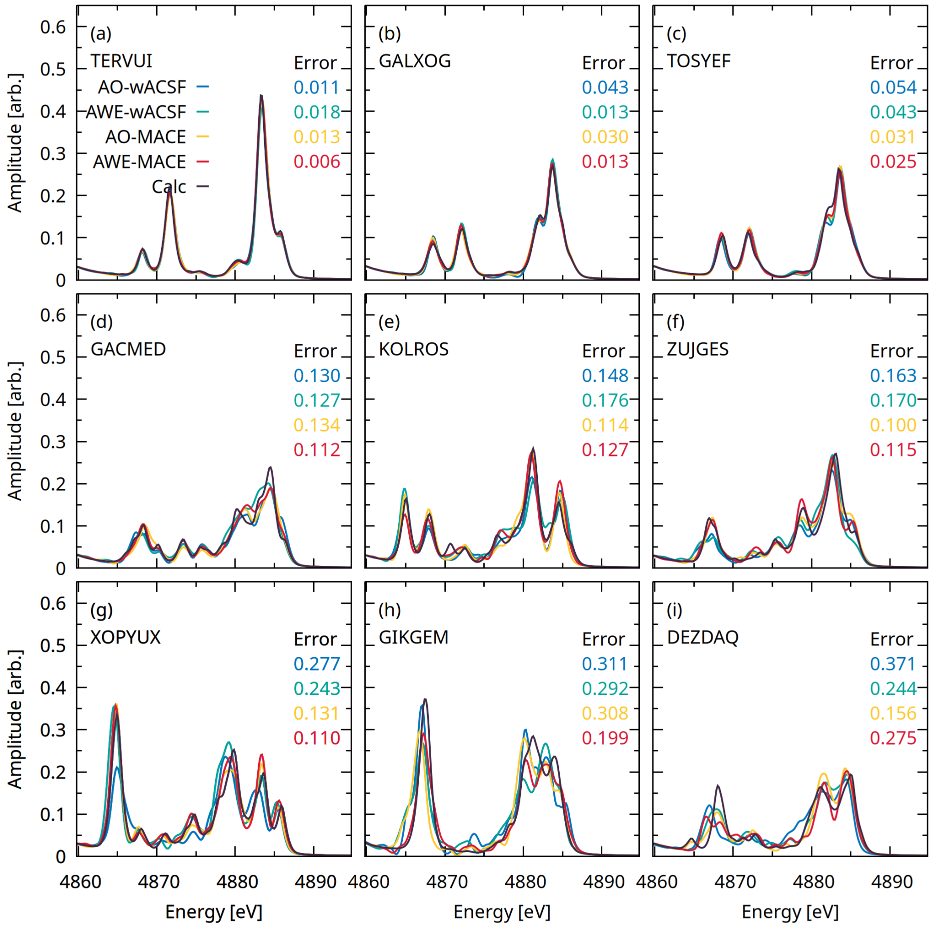

Figure S21: Representative Ti K-edge VtC XES spectra predicted using the AO-wACSF (blue), AWE-wACSF (green), AO-MACE (yellow), and AWE-MACE (red) models. The upper three panels show spectra drawn from the 1st–15th percentiles of the error distribution, corresponding to the best-performing predictions. The central three panels display spectra from the 45th–55th percentiles, representative of median performance, while the lower three panels present spectra from the 85th–100th percentiles, corresponding to the worst performers. The six-character labels in the lower right corner of each panel denote the Cambridge Structural Database (CSD) reference codes for the corresponding samples. The values reported in each panel are the MS-SSIM errors associated with the respective model predictions.

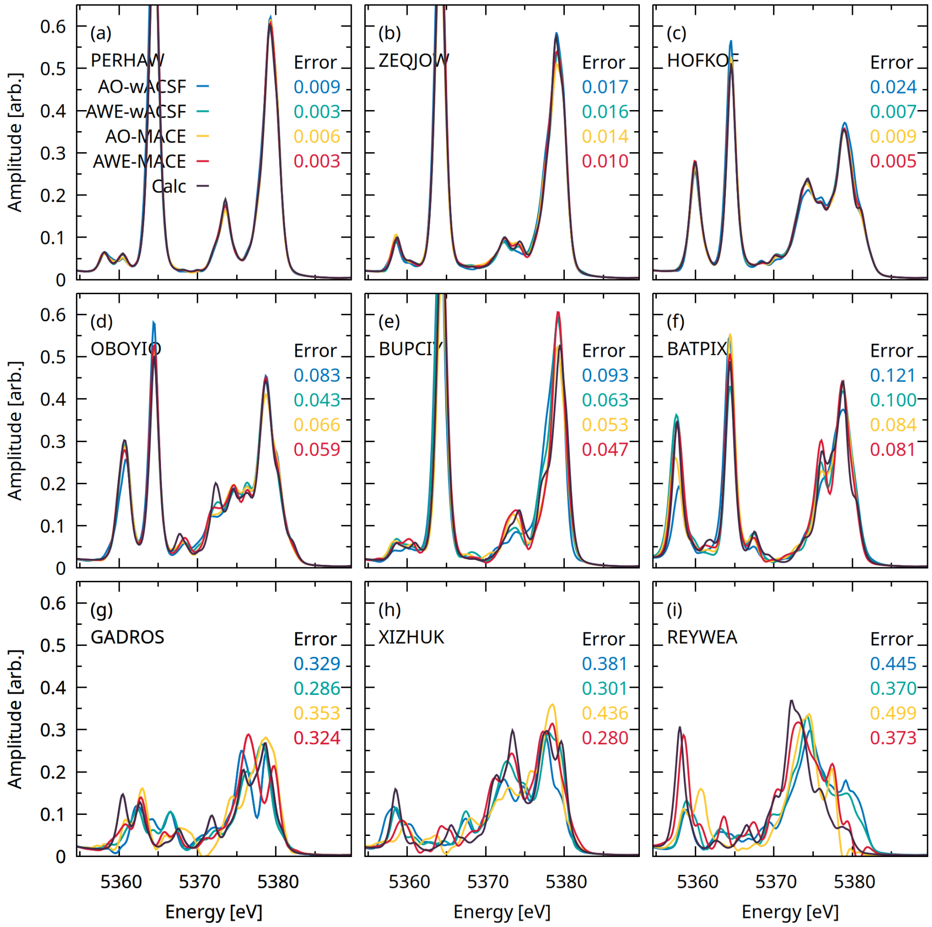

Figure S22: Representative V K-edge VtC XES spectra predicted using the AO-wACSF (blue), AWE-wACSF (green), AO-MACE (yellow), and AWE-MACE (red) models. The upper three panels show spectra drawn from the 1st–15th percentiles of the error distribution, corresponding to the best-performing predictions. The central three panels display spectra from the 45th–55th percentiles, representative of median performance, while the lower three panels present spectra from the 85th–100th percentiles, corresponding to the worst performers. The six-character labels in the lower right corner of each panel denote the Cambridge Structural Database (CSD) reference codes for the corresponding samples. The values reported in each panel are the MS-SSIM errors associated with the respective model predictions.

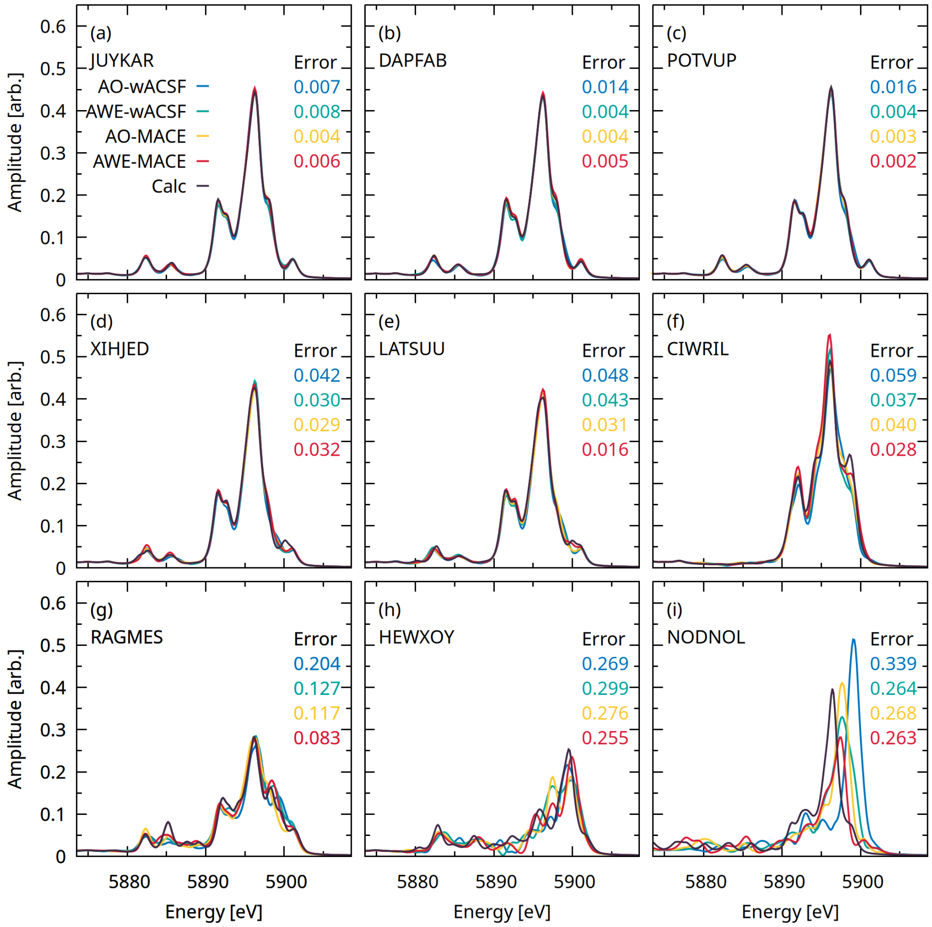

Figure S23: Representative Cr K-edge VtC XES spectra predicted using the AO-wACSF (blue), AWE-wACSF (green), AO-MACE (yellow), and AWE-MACE (red) models. The upper three panels show spectra drawn from the 1st–15th percentiles of the error distribution, corresponding to the best-performing predictions. The central three panels display spectra from the 45th–55th percentiles, representative of median performance, while the lower three panels present spectra from the 85th–100th percentiles, corresponding to the worst performers. The six-character labels in the lower right corner of each panel denote the Cambridge Structural Database (CSD) reference codes for the corresponding samples. The values reported in each panel are the MS-SSIM errors associated with the respective model predictions.

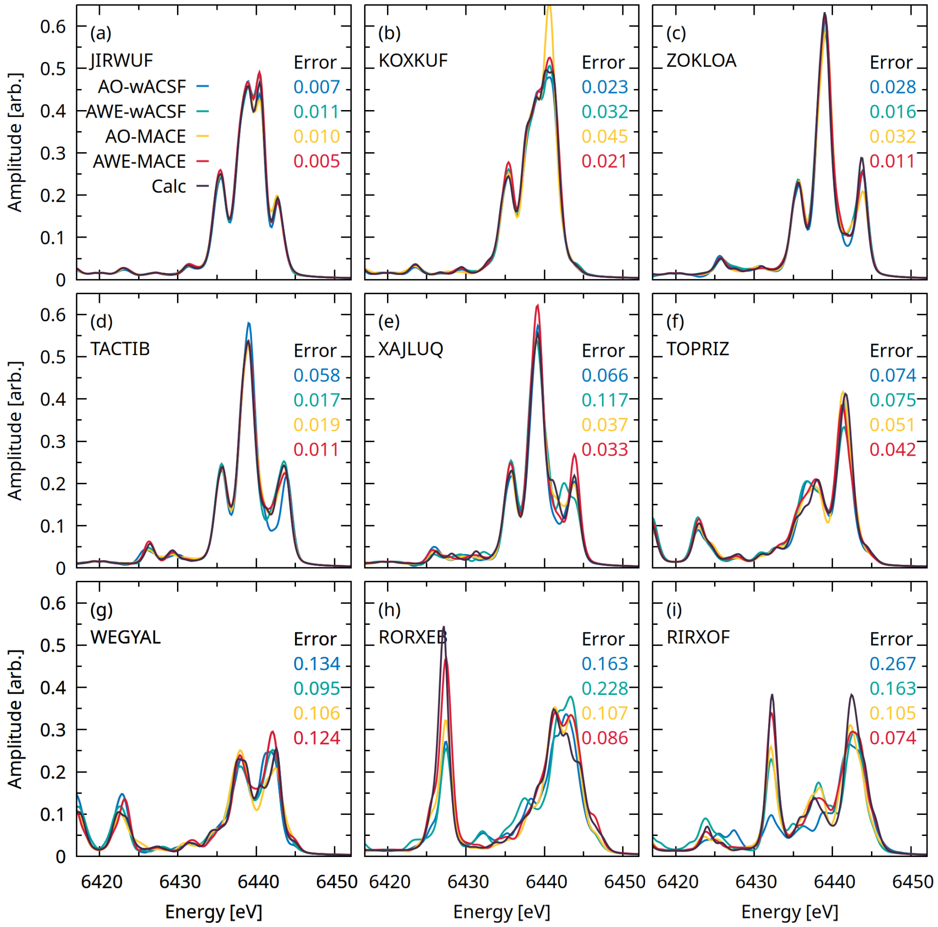

Figure S24: Representative Mn K-edge VtC XES spectra predicted using the AO-wACSF (blue), AWE-wACSF (green), AO-MACE (yellow), and AWE-MACE (red) models. The upper three panels show spectra drawn from the 1st–15th percentiles of the error distribution, corresponding to the best-performing predictions. The central three panels display spectra from the 45th–55th percentiles, representative of median performance, while the lower three panels present spectra from the 85th–100th percentiles, corresponding to the worst performers. The six-character labels in the lower right corner of each panel denote the Cambridge Structural Database (CSD) reference codes for the corresponding samples. The values reported in each panel are the MS-SSIM errors associated with the respective model predictions.

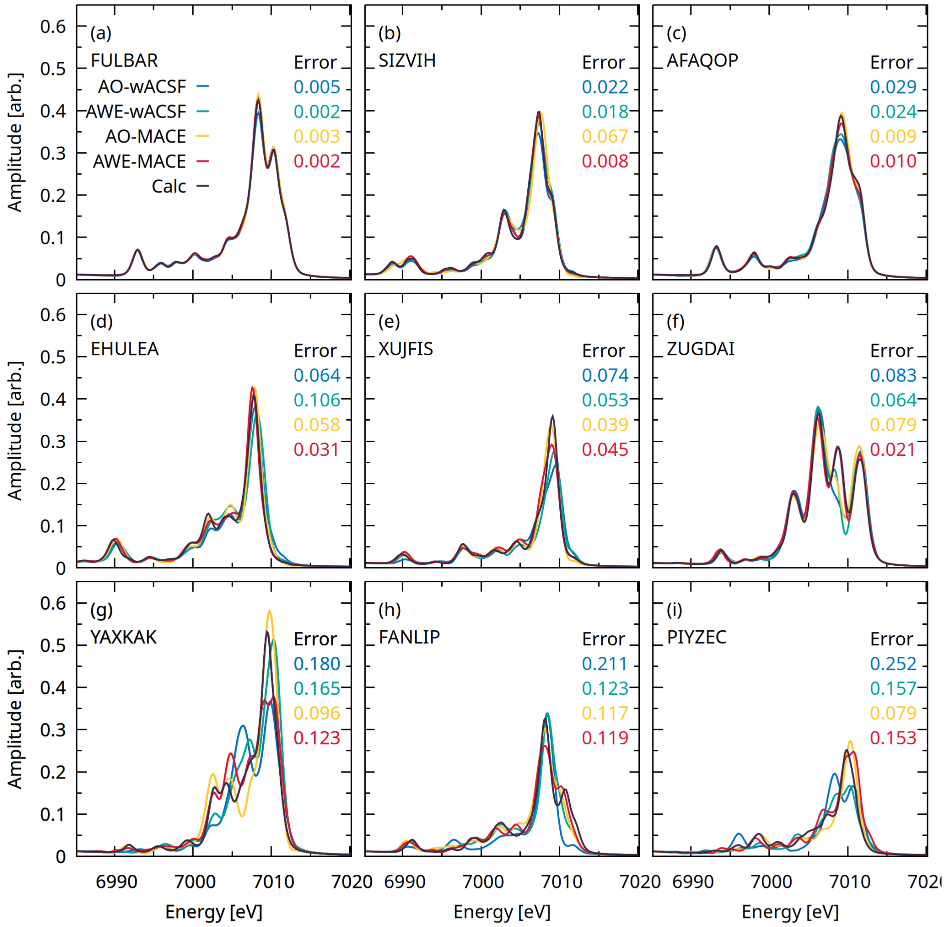

Figure S25: Representative Fe K-edge VtC XES spectra predicted using the AO-wACSF (blue), AWE-wACSF (green), AO-MACE (yellow), and AWE-MACE (red) models. The upper three panels show spectra drawn from the 1st–15th percentiles of the error distribution, corresponding to the best-performing predictions. The central three panels display spectra from the 45th–55th percentiles, representative of median performance, while the lower three panels present spectra from the 85th–100th percentiles, corresponding to the worst performers. The six-character labels in the lower right corner of each panel denote the Cambridge Structural Database (CSD) reference codes for the corresponding samples. The values reported in each panel are the MS-SSIM errors associated with the respective model predictions.

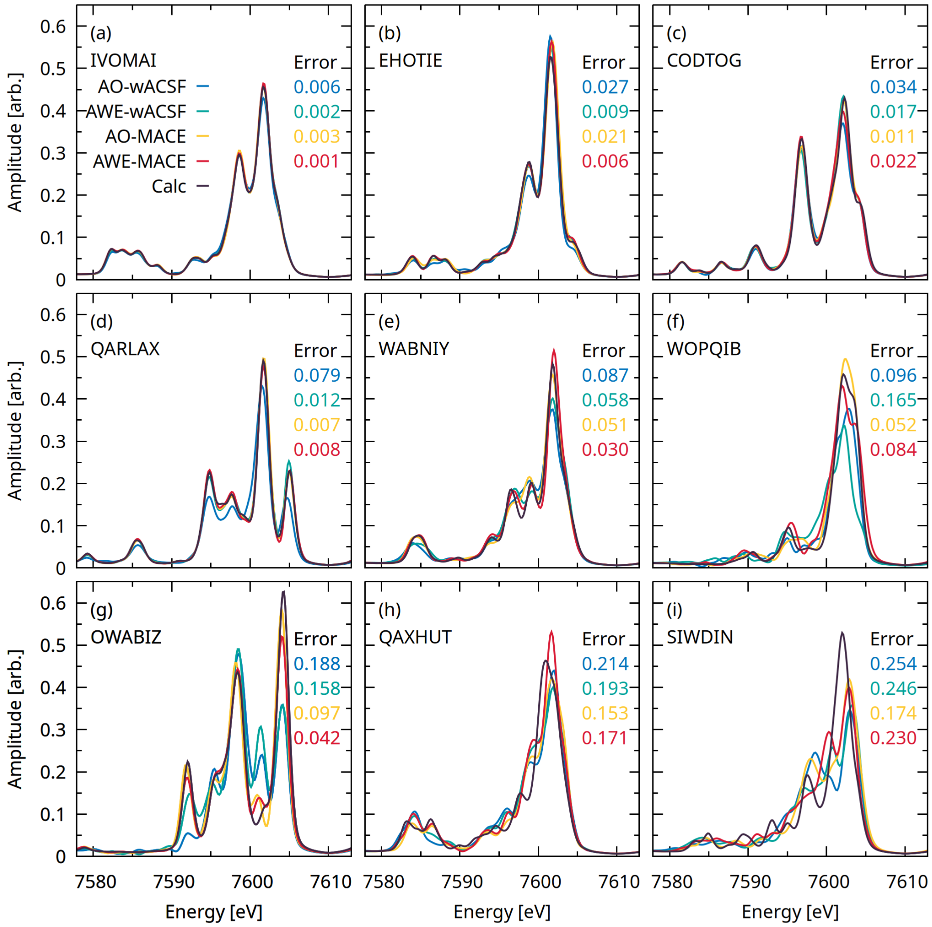

Figure S26: Representative Co K-edge VtC XES spectra predicted using the AO-wACSF (blue), AWE-wACSF (green), AO-MACE (yellow), and AWE-MACE (red) models. The upper three panels show spectra drawn from the 1st–15th percentiles of the error distribution, corresponding to the best-performing predictions. The central three panels display spectra from the 45th–55th percentiles, representative of median performance, while the lower three panels present spectra from the 85th–100th percentiles, corresponding to the worst performers. The six-character labels in the lower right corner of each panel denote the Cambridge Structural Database (CSD) reference codes for the corresponding samples. The values reported in each panel are the MS-SSIM errors associated with the respective model predictions.

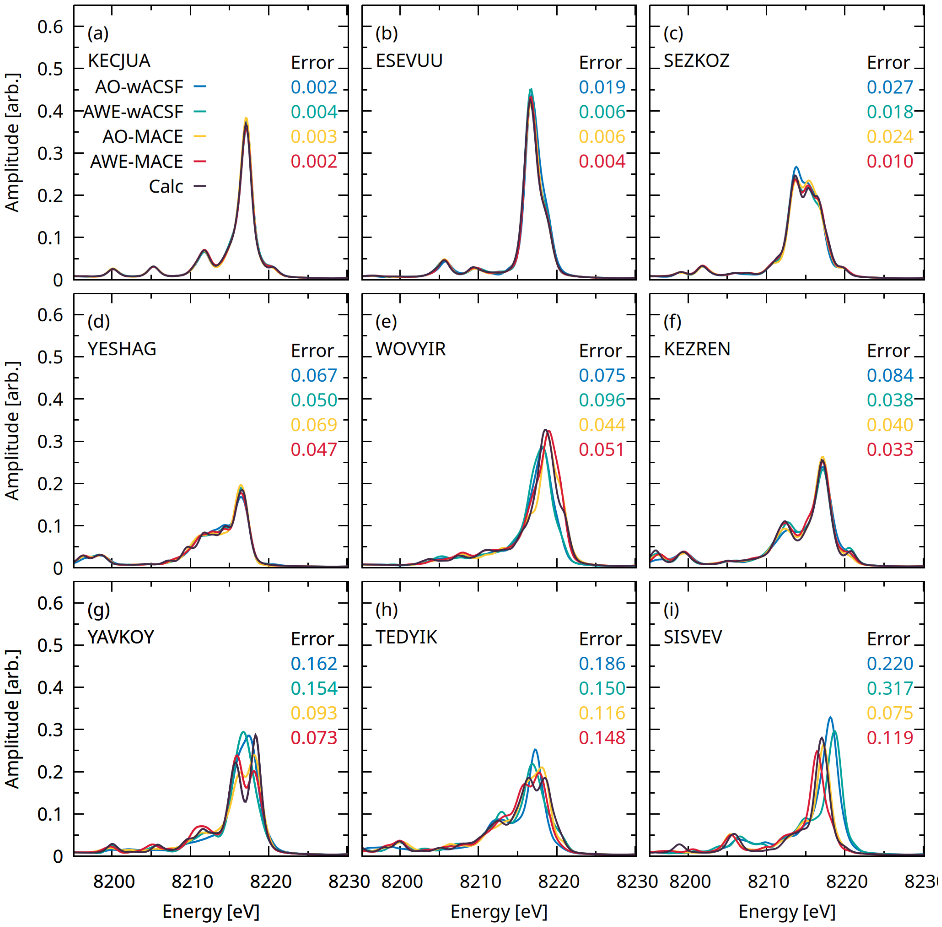

Figure S27: Representative Ni K-edge VtC XES spectra predicted using the AO-wACSF (blue), AWE-wACSF (green), AO-MACE (yellow), and AWE-MACE (red) models. The upper three panels show spectra drawn from the 1st–15th percentiles of the error distribution, corresponding to the best-performing predictions. The central three panels display spectra from the 45th–55th percentiles, representative of median performance, while the lower three panels present spectra from the 85th–100th percentiles, corresponding to the worst performers. The six-character labels in the lower right corner of each panel denote the Cambridge Structural Database (CSD) reference codes for the corresponding samples. The values reported in each panel are the MS-SSIM errors associated with the respective model predictions.

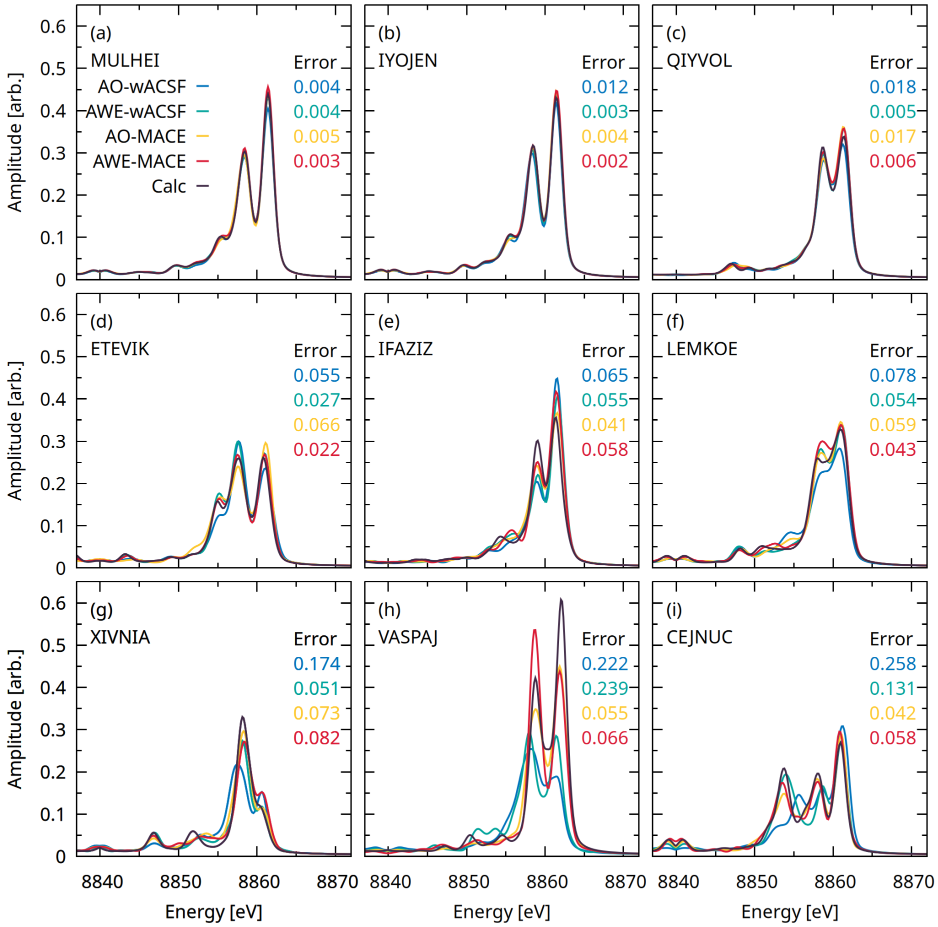

Figure S28: Representative Cu K-edge VtC XES spectra predicted using the AO-wACSF (blue), AWE-wACSF (green), AO-MACE (yellow), and AWE-MACE (red) models. The upper three panels show spectra drawn from the 1st–15th percentiles of the error distribution, corresponding to the best-performing predictions. The central three panels display spectra from the 45th–55th percentiles, representative of median performance, while the lower three panels present spectra from the 85th–100th percentiles, corresponding to the worst performers. The six-character labels in the lower right corner of each panel denote the Cambridge Structural Database (CSD) reference codes for the corresponding samples. The values reported in each panel are the MS-SSIM errors associated with the respective model predictions.

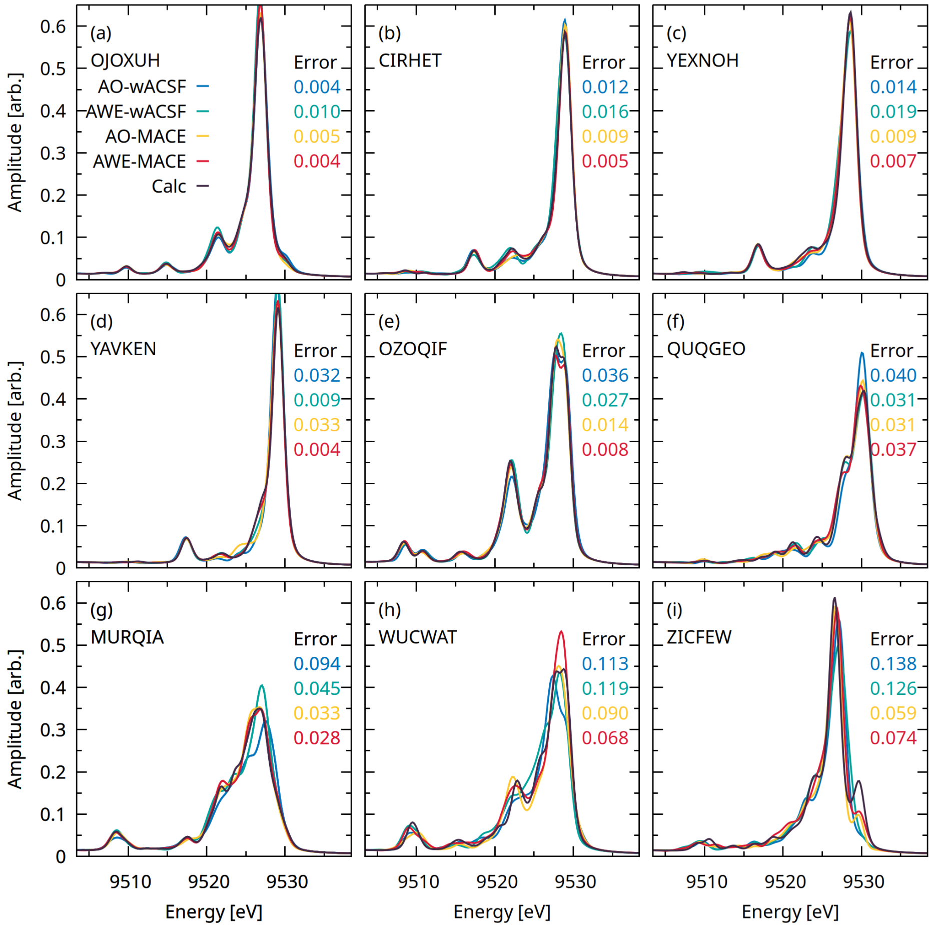

Figure S29: Representative Zn K-edge VtC XES spectra predicted using the AO-wACSF (blue), AWE-wACSF (green), AO-MACE (yellow), and AWE-MACE (red) models. The upper three panels show spectra drawn from the 1st–15th percentiles of the error distribution, corresponding to the best-performing predictions. The central three panels display spectra from the 45th–55th percentiles, representative of median performance, while the lower three panels present spectra from the 85th–100th percentiles, corresponding to the worst performers. The six-character labels in the lower right corner of each panel denote the Cambridge Structural Database (CSD) reference codes for the corresponding samples. The values reported in each panel are the MS-SSIM errors associated with the respective model predictions.

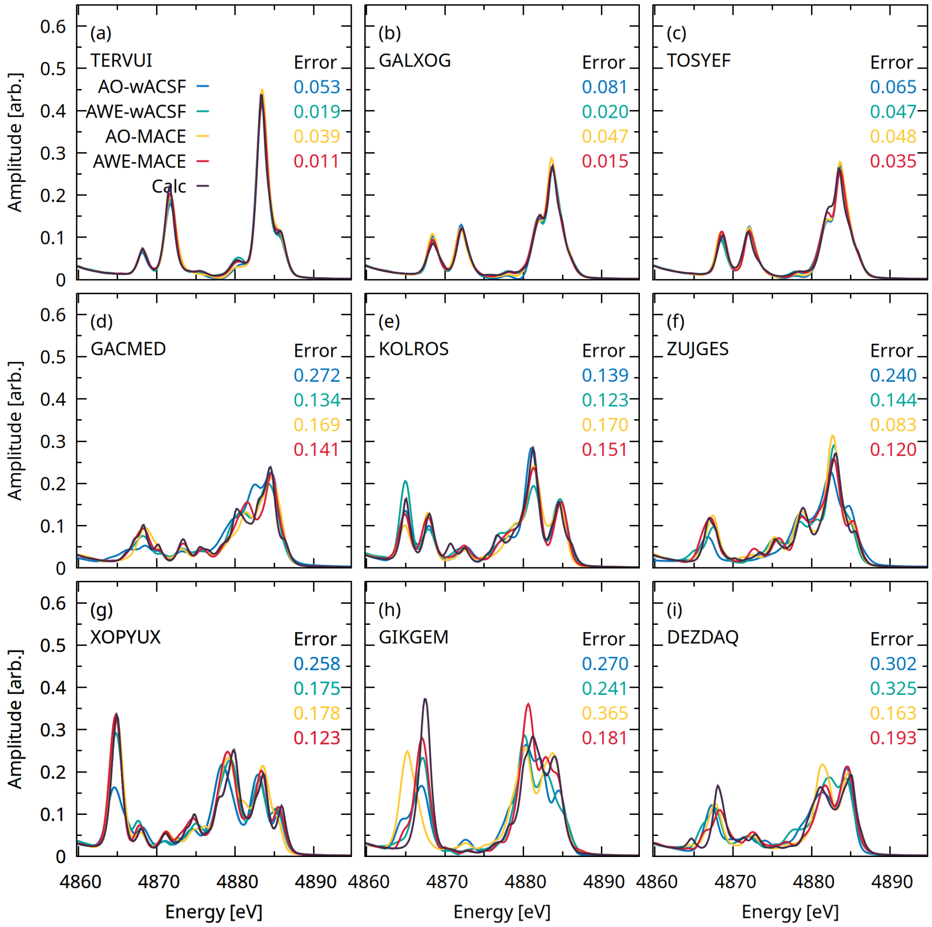

Figure S30: Equivalent plot to S21, showing XAS predictions obtained using a model trained on the combined “ALL”-atom dataset rather than element-specific data.

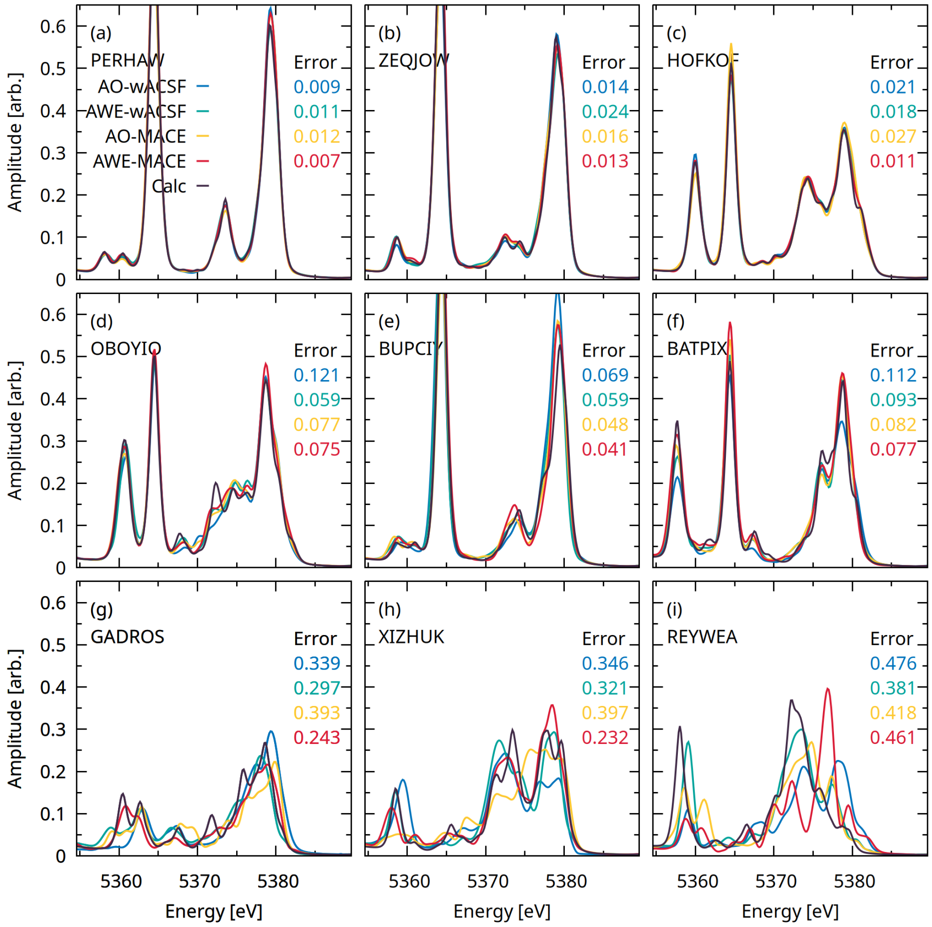

Figure S31: Equivalent plot to S22, showing XAS predictions obtained using a model trained on the combined “ALL”-atom dataset rather than element-specific data.

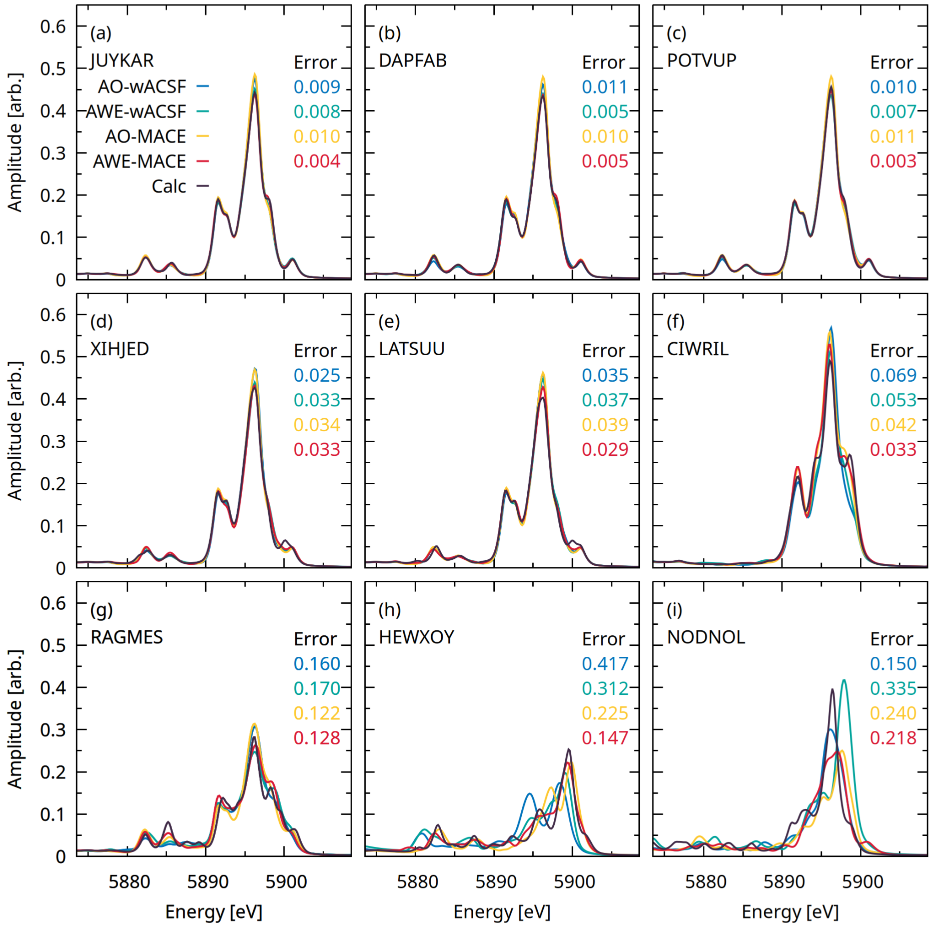

Figure S32: Equivalent plot to S23, showing XAS predictions obtained using a model trained on the combined "ALL"-atom dataset rather than element-specific data.

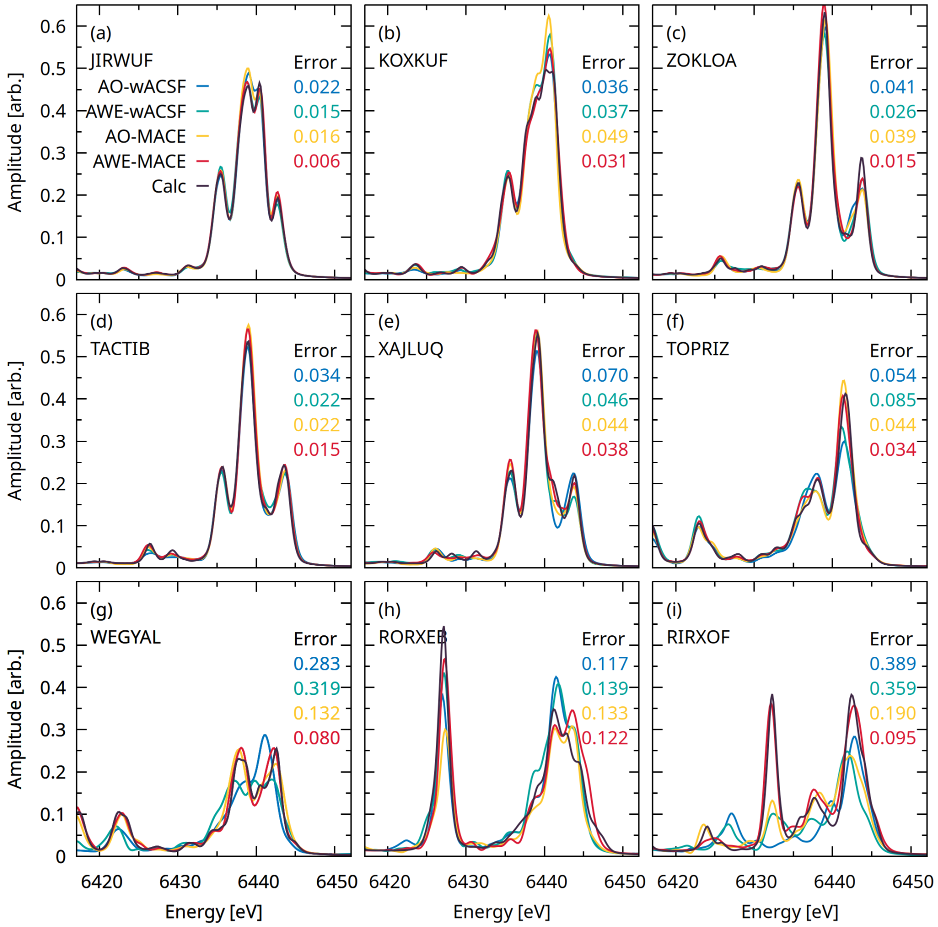

Figure S33: Equivalent plot to S24, showing XAS predictions obtained using a model trained on the combined "ALL"-atom dataset rather than element-specific data.

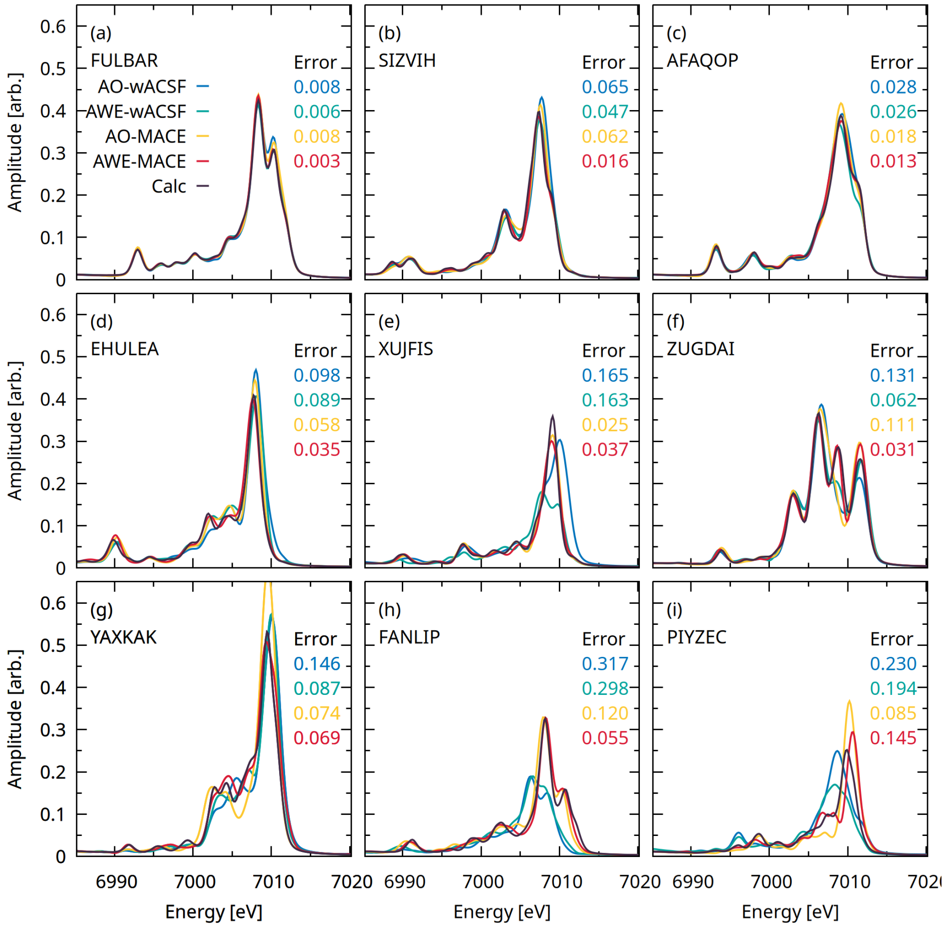

Figure S34: Equivalent plot to S25, showing XAS predictions obtained using a model trained on the combined "ALL"-atom dataset rather than element-specific data.

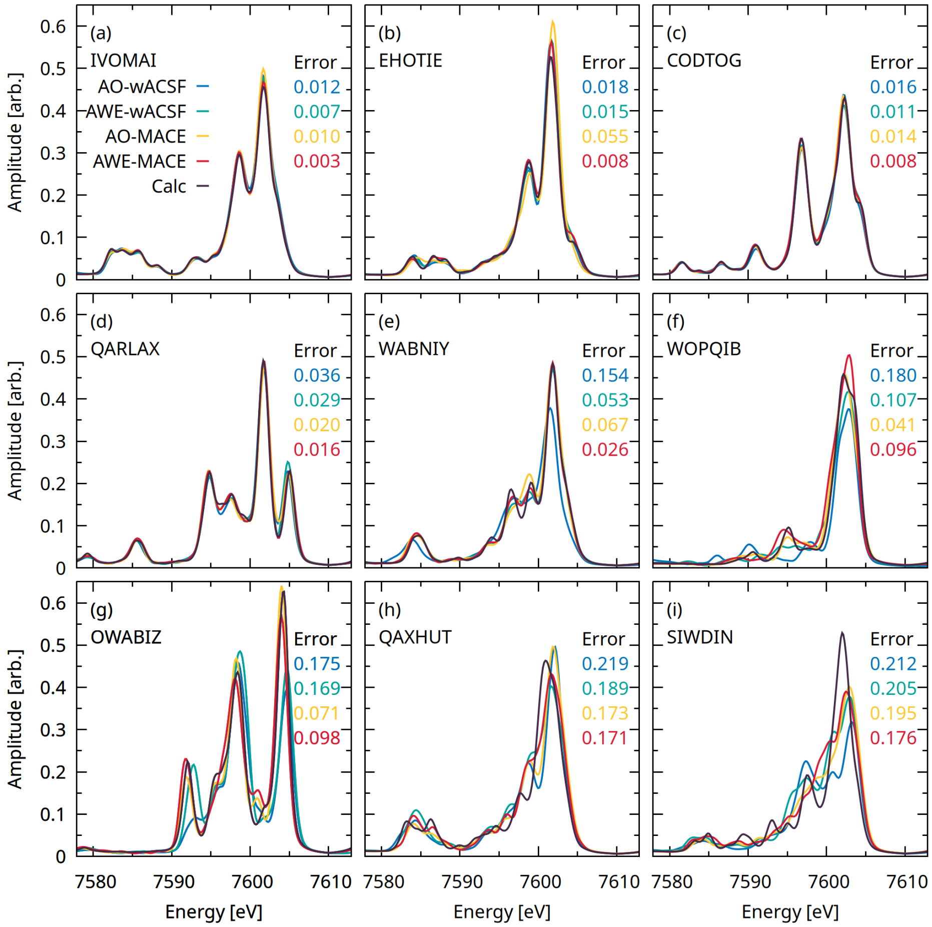

Figure S35: Equivalent plot to S26, showing XAS predictions obtained using a model trained on the combined “ALL”-atom dataset rather than element-specific data.

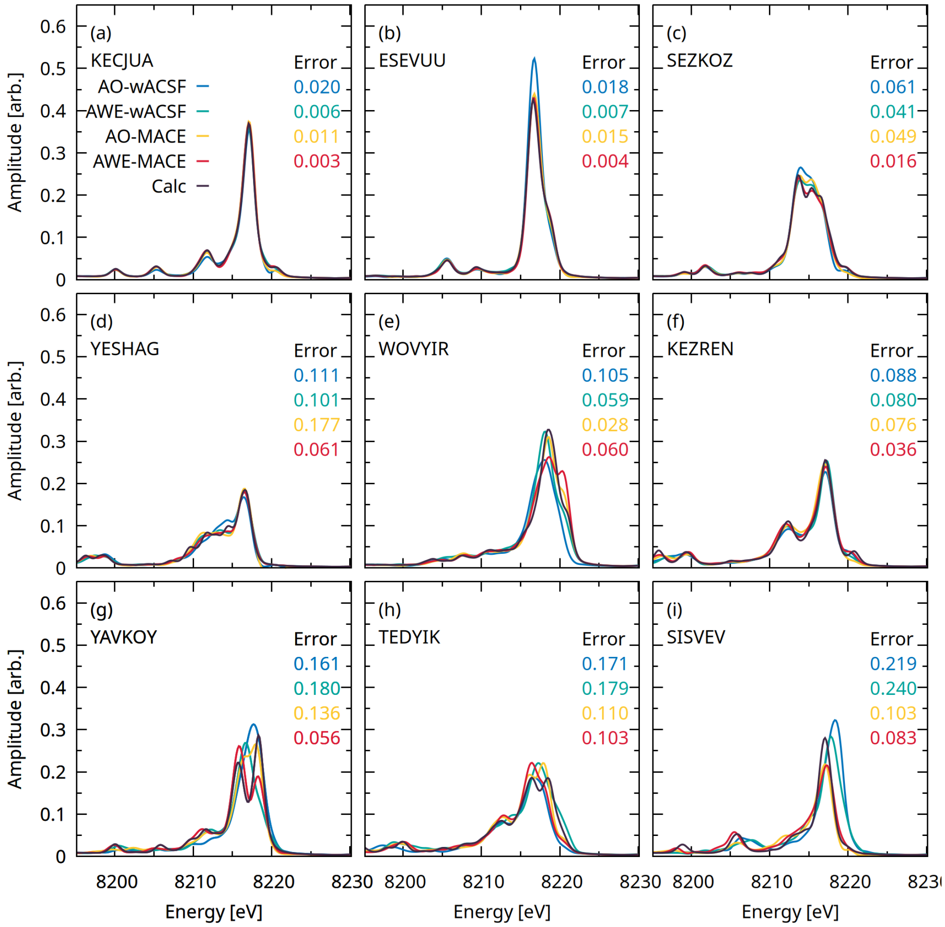

Figure S36: Equivalent plot to S27, showing XAS predictions obtained using a model trained on the combined “ALL”-atom dataset rather than element-specific data.

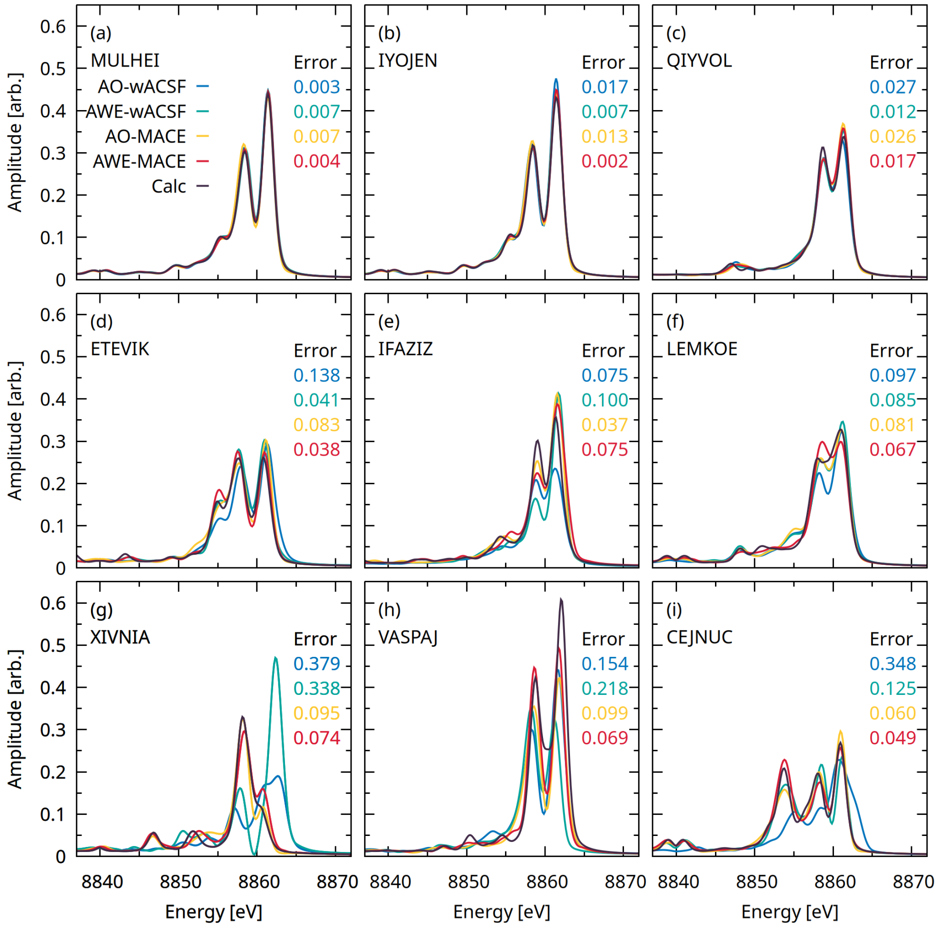

Figure S37: Equivalent plot to S28, showing XAS predictions obtained using a model trained on the combined "ALL"-atom dataset rather than element-specific data.

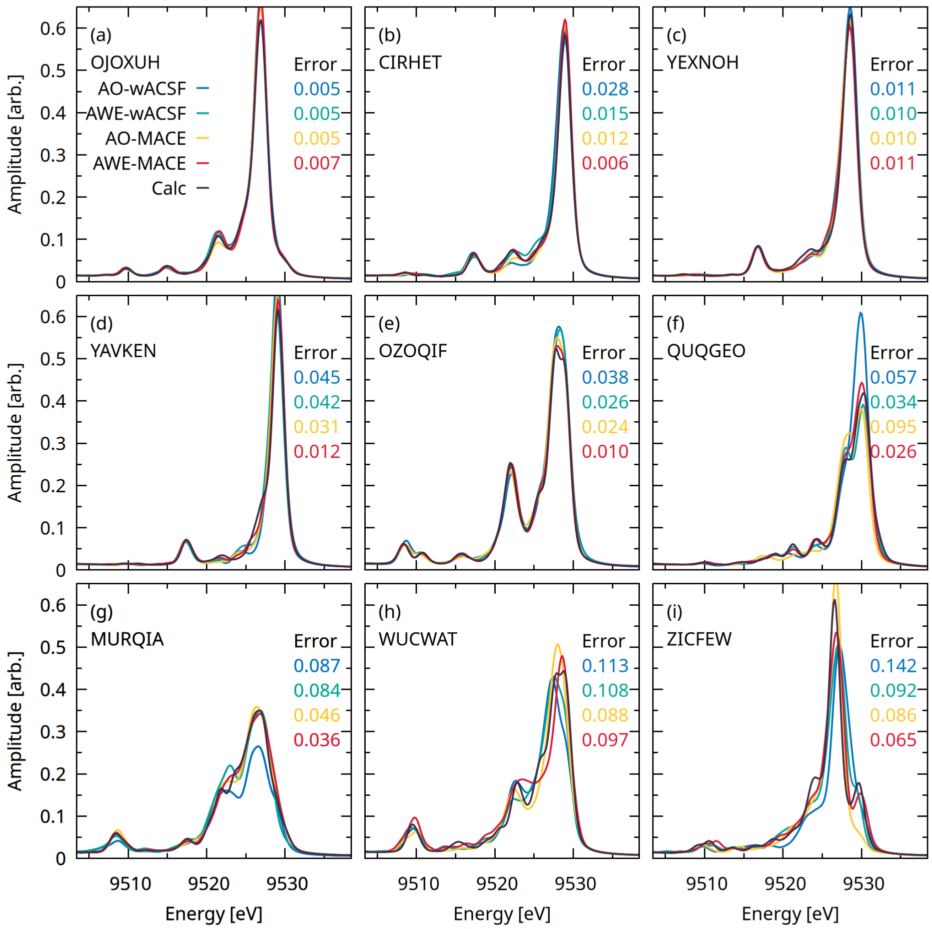

Figure S38: Equivalent plot to S29, showing XAS predictions obtained using a model trained on the combined "ALL"-atom dataset rather than element-specific data.

Sensitivity to Compositional Variation at Zn K-edge

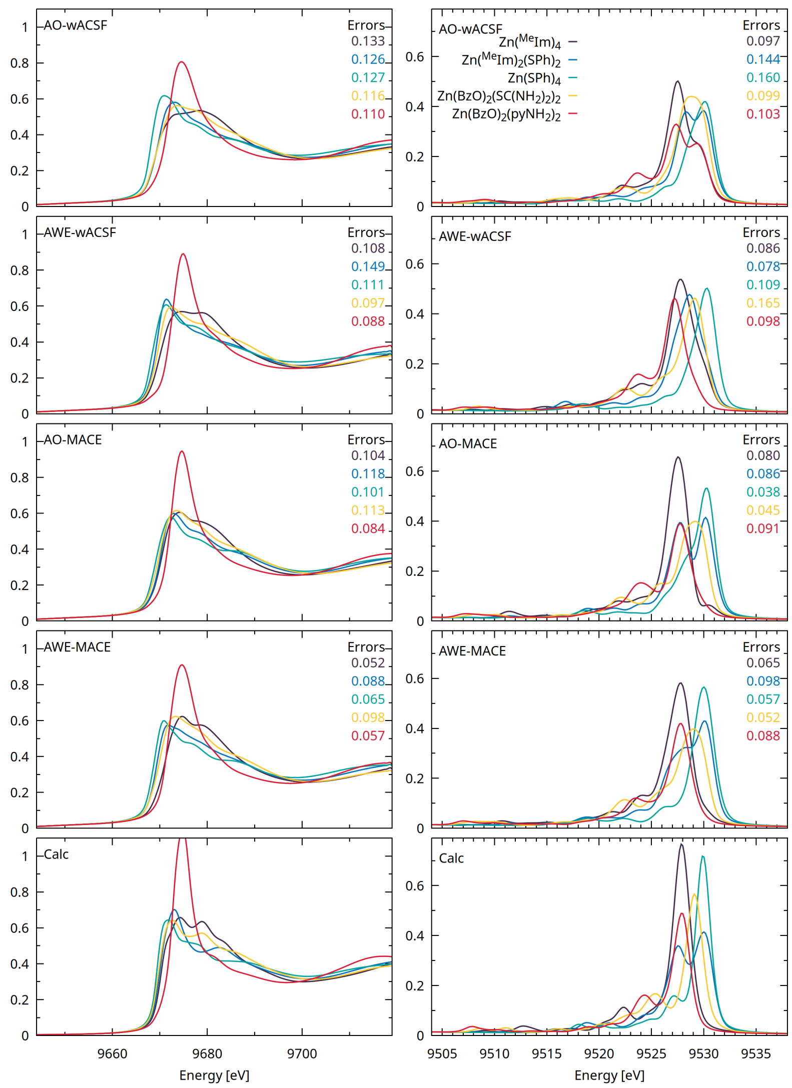

Figure S39: XAS and XES spectra of $[Zn(^{Me}Im)_4]^{2+}$, $[Zn(^{Me}Im)_2(SPh)_2]$, $[Zn(SPh)_4]^{2-}$, $[Zn(BzO)_2(SC(NH_2)_2)_2]$, and $[Zn(BzO)_2(pyNH_2)_2]$. Spectra are shown for the reference quantum-chemical calculations and for predictions obtained using the AO-wACSF, AO-MACE, AWE-wACSF and AWE-MACE models. Insets report the MS-SSIM errors between the calculated and predicted spectra for each complex.

Sensitivity to Geometric Variation at Fe K-edge

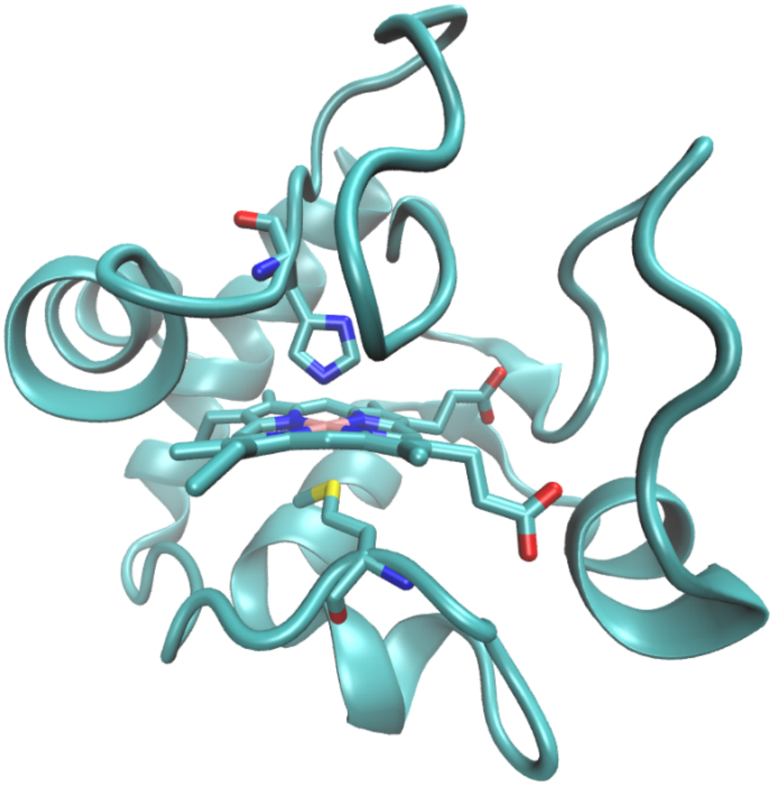

Figure S40: Figure of the cytochrome *c* protein, with the porphyrin active site highlighted with atomic resolution.

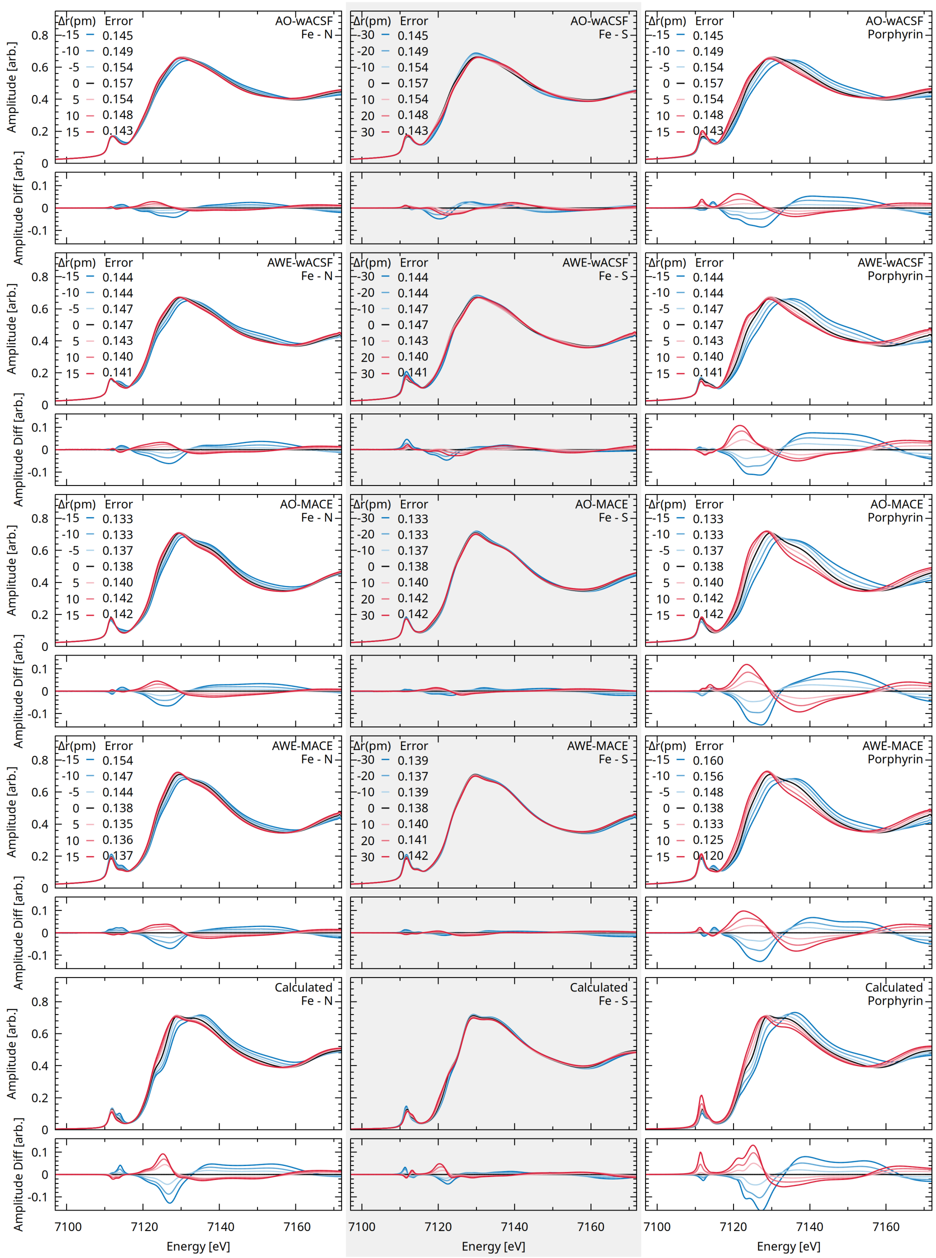

Figure S41: XAS spectral evolution under systematic structural perturbations of the Fe centre. The columns show variations in (i) the Fe–N_{His} distance to the proximal histidine ligand (left), (ii) the Fe–S distance to the distal methionine ligand (middle), and (iii) the Fe–N_p distances associated with the four pyrrolic nitrogen atoms of the porphyrin macrocycle (right). For each perturbation, the corresponding spectrum at the equilibrium geometry is shown beneath the distorted-geometry spectra for reference. The rows report predictions obtained using, from top to bottom: AO–wACSF, AWE–wACSF, AO–MACE, and AWE–MACE models, with the final row showing the reference calculated spectra..

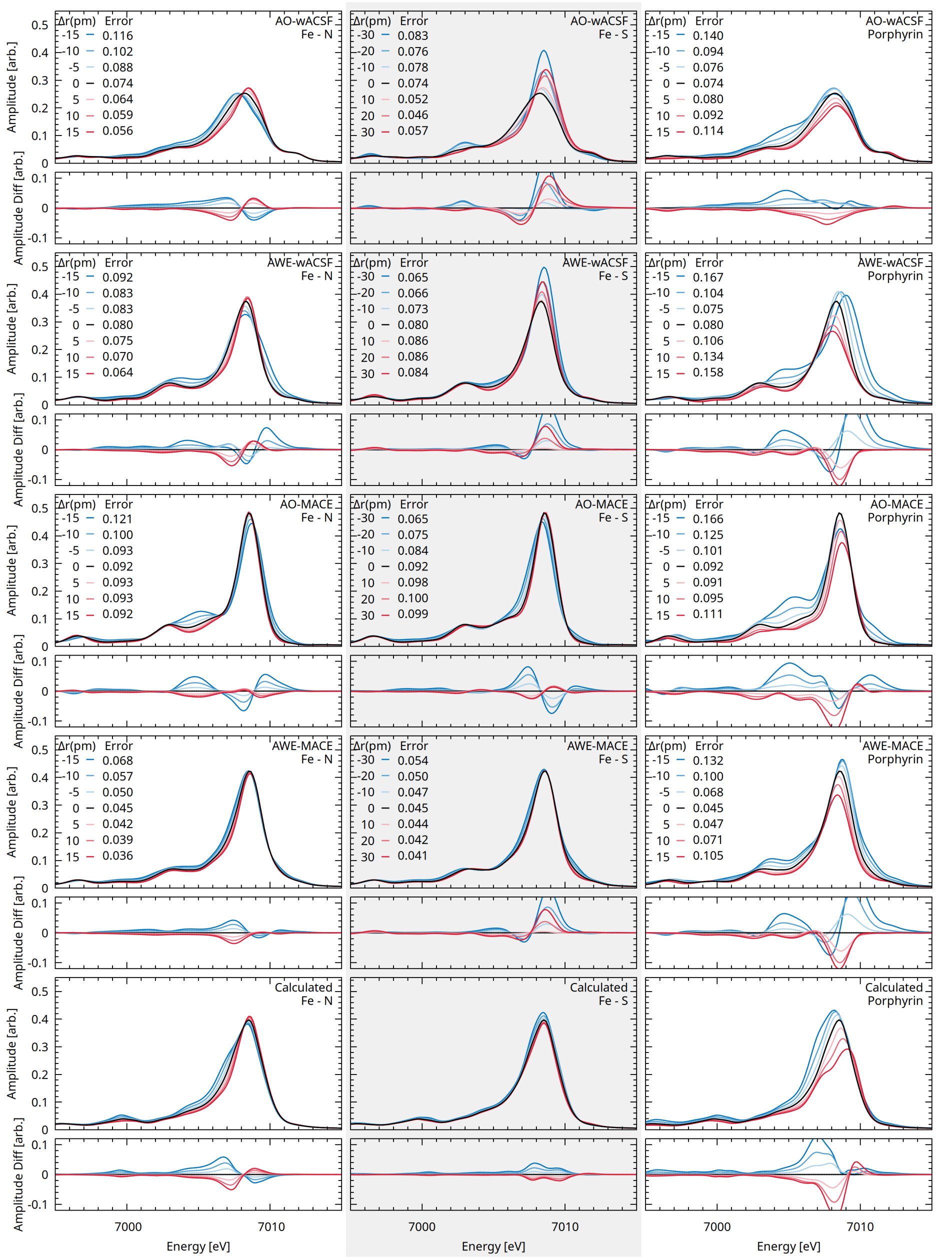

Figure S42: XES spectral evolution under systematic structural perturbations of the Fe centre. The columns show variations in (i) the Fe–N_{His} distance to the proximal histidine ligand (left), (ii) the Fe–S distance to the distal methionine ligand (middle), and (iii) the Fe–N_p distances associated with the four pyrrolic nitrogen atoms of the porphyrin macrocycle (right). For each perturbation, the corresponding spectrum at the equilibrium geometry is shown beneath the distorted-geometry spectra for reference. The rows report predictions obtained using, from top to bottom: AO–wACSF, AWE–wACSF, AO–MACE, and AWE–MACE models, with the final row showing the reference calculated spectra.
